# Embigin is a fibronectin receptor that affects sebaceous gland differentiation and metabolism

**DOI:** 10.1016/j.devcel.2022.05.011

**Published:** 2022-06-20

**Authors:** Kalle Sipilä, Emanuel Rognoni, Johanna Jokinen, Mukul Tewary, Matteo Vietri Rudan, Salli Talvi, Ville Jokinen, Käthe M. Dahlström, Kif Liakath-Ali, Atefeh Mobasseri, Xinyi Du-Harpur, Jarmo Käpylä, Stephen L. Nutt, Tiina A. Salminen, Jyrki Heino, Fiona M. Watt

**Affiliations:** 1 Centre for Gene Therapy & Regenerative Medicine, King’s College London, London SE1 9RT, UK; 2 Centre for Cell Biology and Cutaneous Research, Blizard Institute, Faculty of Medicine and Dentistry, Queen Mary University of London, London E1 2AT, UK; 3 Department of Life Technologies, University of Turku, Turku 20014, Finland; 4 Structural Bioinformatics Laboratory, InFLAMES Research Flagship Center, Faculty of Science and Engineering, Åbo Akademi University, Turku 20520, Finland; 5 Department of Molecular and Cellular Physiology, Stanford University, Stanford, CA 94305, USA; 6 The Walter and Eliza Hall Institute of Medical Research, 1G Royal Parade, Parkville, VIC 3052, Australia; 7 Department of Medical Biology, University of Melbourne, Parkville, VIC 3010, Australia; 8 European Molecular Biology Laboratory, Heidelberg 69117, Germany; 9 The Francis Crick Institute, London NW1 1AT, UK

**Keywords:** stem cell niche, cell adhesion, ECM organization, integrin, lipid metabolism, fibronectin, SG differentiation

## Abstract

Stem cell renewal and differentiation are regulated by interactions with the niche. Although multiple cell populations have been identified in distinct anatomical compartments, little is known about niche-specific molecular factors. Using skin as a model system and combining single-cell RNA-seq data analysis, immunofluorescence, and transgenic mouse models, we show that the transmembrane protein embigin is specifically expressed in the sebaceous gland and that the number of embigin-expressing cells is negatively regulated by Wnt. The loss of embigin promotes exit from the progenitor compartment and progression toward differentiation, and also compromises lipid metabolism. Embigin modulates sebaceous niche architecture by affecting extracellular matrix organization and basolateral targeting of monocarboxylate transport. We discover through ligand screening that embigin is a direct fibronectin receptor, binding to the N-terminal fibronectin domain without impairing integrin function. Our results solve the long-standing question of how embigin regulates cell adhesion and demonstrate a mechanism that couples adhesion and metabolism.

## Highlights

• Embigin (EMB) marks Wnt-regulated, lipid-producing, sebaceous gland cells
• EMB supports adhesion to ECM via direct binding to the N-terminal part of fibronectin
• EMB deletion drives sebocyte progenitors from basal layer to suprabasal differentiation
• EMB regulates stiffness, ECM composition, and MCT1 localization in sebaceous glands

## Graphical abstract

**Figure:**
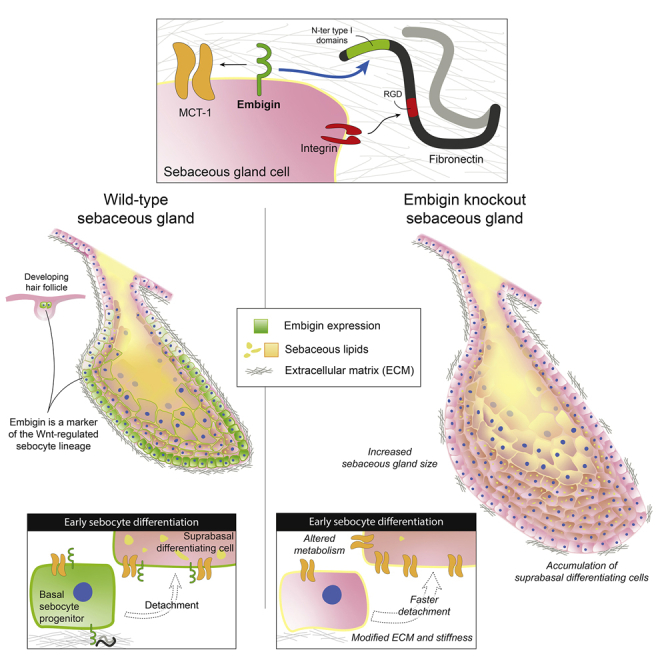

## Teaser

Extracellular matrix (ECM) interaction is an important regulator of epithelial stem cells and progenitors. Sipilä et al. report a mechanism by which embigin directly binds to fibronectin and mediates progenitor cell anchoring to the ECM, regulating the differentiation and metabolism of sebocytes as well as the stiffness of the sebaceous gland microenvironment.

## Introduction

The self-renewal and differentiation of stem cells are critically regulated via interactions with their local microenvironment, referred to as the “niche,” in normal homeostasis and in response to injuries (Chacón-Martínez et al., 2018; Hoggatt et al., 2016; Watt and Hogan, 2000). Niches are unique and specific to different stem cell populations, ensuring the optimal maintenance of stem renewal and differentiation within a tissue. The features of the stem cell niche include extracellular matrix (ECM) components as well as homologous and heterologous cell-cell contacts (Lane et al., 2014). Cell-cell contacts involve multiple tightly regulated, secreted, and membrane-bound growth factors and cytokines. The ECM can function as a reservoir of signaling factors, including TGF-βs (Hynes, 2009), but it also regulates physical parameters such as the stiffness and anatomical shape of the niche (Watt, 2016).

Skin provides an excellent model system to study how different stem cells and progenitors interact with their niches (Watt and Fujiwara, 2011). Recent advances in single-cell RNA-sequencing (scRNA-seq) technologies have enormously increased our understanding of different cell populations in skin and other tissues (Joost et al., 2020; Wilbrey-Clark et al., 2020). At the same time, scRNA-seq has become a powerful tool for characterizing the molecular determinants of cell-niche crosstalk (Silberstein et al., 2016), including the large-scale analysis of extracellular receptor-ligand pairs in biologically relevant tissue contexts (Vento-Tormo et al., 2018). However, due to the challenges of high-throughput identification of novel extracellular receptor-ligand pairs (Chong et al., 2018), a more individualized approach is often required when the interaction partners for membrane proteins or secreted factors are not known.

In this work, based on the analysis of published scRNA-seq data (Joost et al., 2016), we identified embigin (EMB) as a potential epithelial niche interacting factor in mouse skin that is specifically expressed in sebaceous glands (SGs). EMB is a type I transmembrane receptor that belongs to the immunoglobulin superfamily in a subgroup with basigin and neuroplastin, but it has remained a less-studied member of this protein family (Muramatsu and Miyauchi, 2003). Recently, EMB has been shown to be a component of the hematopoietic stem cell niche, where it is upregulated in the osteo lineage niche cells that are in close proximity to the transplanted hematopoietic stem and progenitor cells (HSPCs) (Silberstein et al., 2016). EMB regulates the quiescence and homing of HSPCs, but the exact mechanism whereby stem cell behavior is regulated by EMB has not been reported. Here, we show the direct molecular mechanism by which EMB regulates cell behavior, linking cell adhesion and metabolite transport in epithelial progenitor cells.

## Results

### EMB is a marker of the lipid-producing sebocyte lineage that is negatively regulated by Wnt

**Figure 1:**
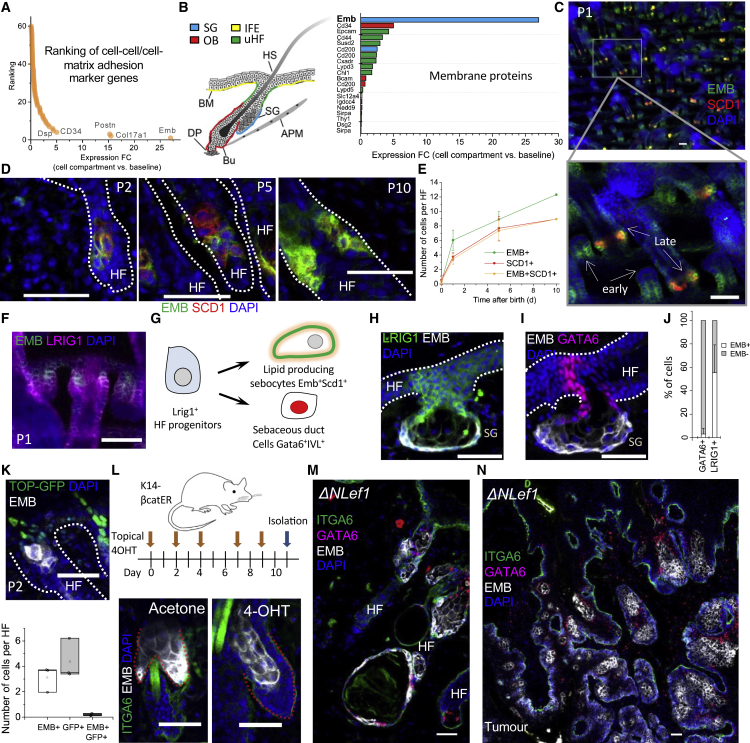
Identification of embigin as a sebocyte marker regulated by Wnt (A and B) Ranking of epithelial marker genes, identified by Joost et al. (Joost et al., 2016) and filtered by GO terms for cell-cell adhesion mediator (GO:0098632) and cell-matrix adhesion (GO:0007160) (A). Non-integrin membrane proteins are shown in a separate graph with schematic of different epidermal regions (B). (C) A mouse tail wholemount (P1) stained with antibodies to EMB and lipid synthesis marker SCD1. (D) Cryosections of neonatal mouse back skin collected at days P2, P5, and P10 and labeled with antibodies against SCD1 and EMB. (E) Quantification of EMB+ and SCD1+ cells during mouse skin development. The average number of cells per HF ± SD is shown. n = 17 P0 (from 2 mice), 25 P2 (from 3 mice), 29 P5 (from 3 mice), and 16 P10 (from 2 mice) HFs were analyzed. (F) P1 tail epidermal wholemount stained with antibodies against EMB and stem cell marker LRIG1. (G) Schematic of SG lineages. (H and I) Cryosections of adult mouse tail skin stained with antibodies against EMB (gray), LRIG1 (green) (H), and the SG duct marker GATA6 (red) (I). (J) Quantification of EMB+GATA6+ and EMB+LRIG1+ double-positive cells in the upper hair follicle. Average % ± SD is shown. A total number of n = 542 GATA6+ cells and 1,069 LRIG1+ cells from 3 mice were analyzed. (K) Cryosection of back skin of TOP-GFP reporter (green) mouse (P2) stained with EMB antibody (gray) and quantification of single- and double-positive cells in the upper hair follicle. Average ± SD of mice shown. n = 83 hair follicles from 3 mice (P1–P2) were analyzed. (L) Adult telogen K14βcatER mice were treated with acetone or 4OH-tamoxifen on the days shown. The skin was then isolated and stained with antibodies against EMB (gray) and basal cell marker ITGA6 (green). Basement membrane is denoted by red dotted line. (M and N) Cryosections from abnormal hair follicles (M) and sebaceous tumor (N) of ΔNLef1 mouse back skin labeled with antibodies against EMB (red) and GATA6 (magenta). (C, D, F, H, I, and K–N) DAPI nuclear stain (blue). All scale bars: 50 μm.

To discover novel niche interacting factors in skin, we analyzed published scRNA-seq data from the adult mouse epidermis (Joost et al., 2016) by using marker genes that are annotated in Gene Ontology (GO) as encoding ECM proteins or proteins interacting with the ECM (Figures 1A, 1B, and S1A). We ranked the genes according to fold change between a specific compartment and the basal level of all the analyzed cells. We identified multiple genes that were selectively upregulated in specific cell populations in contact with the basement membrane. These genes included nephronectin (*Npnt*) and periostin (*Postn*), upregulated in the outer bulge of the hair follicle (HF) (Fujiwara et al., 2011; Watt and Fujiwara, 2011), as well as the distinct laminin (LN) isoforms in the interfollicular epidermis (IFE) and upper HF (Morgner et al., 2015), which validated our approach.

The most distinct, previously uncharacterized, candidate was EMB, which showed a high enrichment in the SG compartment (Figures 1A and 1B). The SG is a sac-like structure, comprising a proliferative basal layer of cells that give rise to differentiating sebocytes in the center of the gland. As they accumulate lipids, differentiating sebocytes enlarge and eventually burst, releasing sebum, which flows through the sebaceous duct (SD) and lubricates the HF and IFE (Cottle et al., 2013).

To elucidate the role of EMB in the development and homeostasis of SGs, we investigated the expression of EMB in early skin development and in adult SGs (Figures 1C–1J). Although EMB was not expressed in the primitive stages of HF development (stages I–II), we could start to detect low basal EMB expression in the upper part of early HFs (stage III) (Figures 1C and S1B). At this stage, lipid-producing sebocytes had not yet formed, as indicated by the lack of expression of stearoyl-CoA desaturase 1 (SCD1), an enzyme involved in fatty acid synthesis (Figures 1C and 1D). At the later stages of HF development, we could detect the presence of cells that were double positive for EMB and SCD1 expression, characteristic of mature sebocytes (Figures 1C and 1D). The number of these double-positive cells increased as the size of the SG expanded in early postnatal life (Figure 1E).

During development, EMB-positive cells co-expressed the junctional zone stem cell marker LRIG1 (Figures 1F and 1G). EMB was highly expressed in adult SG cells but not in HFs or the IFE (Figures 1G–1J and S1D). Although most adult SG cells were also positive for LRIG1, the SG could be clearly divided into upper GATA6-positive and lower EMB-positive compartments (Figures 1G–1J). This supports the recent finding that the SD lineage is distinct from the rest of the SG, with GATA6 maintaining the SD (Donati et al., 2017; Oulès et al., 2019). We did not detect EMB protein in other skin locations by antibody staining (Figure S1D), even though scRNA-seq indicates that EMB is expressed by macrophages (Joost et al., 2020).

Wnt signaling is a negative regulator of sebocyte fate: Wnt activation by the stabilization of β-catenin leads to the conversion of SGs into HFs (Lo Celso et al., 2004; Silva-Vargas et al., 2005), whereas the inhibition of Wnt-regulated transcription by a dominant-negative mutation of the transcription factor Lef1 promotes sebaceous differentiation (Niemann et al., 2002). Consistent with these observations, EMB-positive cells in developing SGs (P2) were negative for Wnt signaling, as measured by the lack of the expression of H2BeGFP under the control of multiple Lef1/TCF binding sites (Figure 1K). In addition, the activation of β-catenin in the mouse epidermis led to the loss of EMB+ basal cells (Figure 1L). Conversely, K14DNLef1 mice, which express a dominant-negative Lef1 transgene via the K14 promoter, showed EMB expression in the lower pilosebaceous unit where sebaceous lineage cells are not normally present (Figure 1M). In addition, spontaneous sebaceous tumors of K14ΔNLef1 mice expressed high levels of EMB (Figure 1N). GATA6+ cells in abnormal K14ΔNLef1 HFs and tumors did not express EMB (Figures 1M and 1N).

In summary, our results show that EMB is a component of the lipid-producing sebocyte lineage that arises from LRIG1+ HF progenitors during the development of the pilosebaceous unit.

### EMB modulates the transition from progenitor cell to differentiated sebocyte in the SG

**Figure 2:**
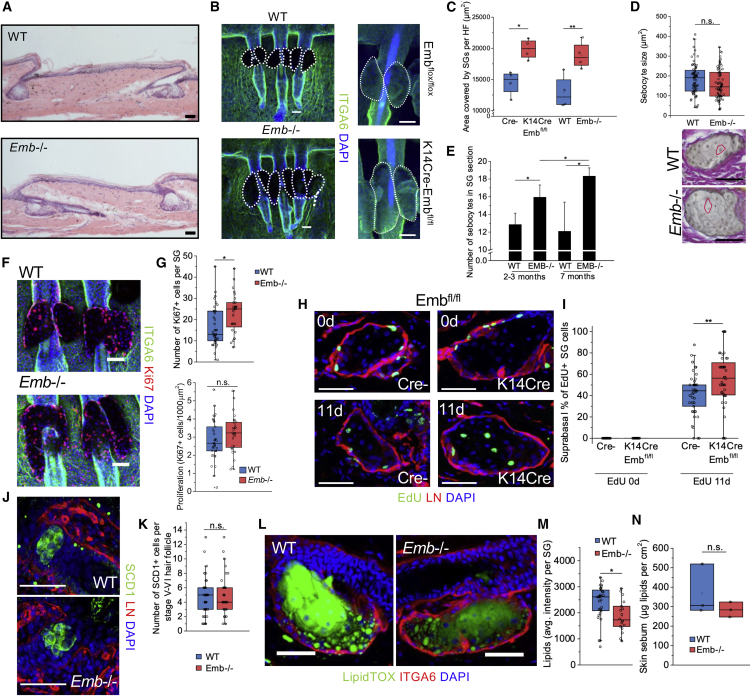
Deletion of embigin disrupts sebaceous gland homeostasis (A) Hematoxylin and eosin staining of the tail skin of adult WT and *Emb*^*−/−*^ mice. (B) Adult tail wholemounts from WT and *Emb*^*−/−*^ as well as from Emb^flox/flox^ and K14CreEmb^flox/flox^ mice labeled with antibodies against ITGA6 (green) and DAPI (blue). (C) Quantification of area covered by SGs per HF. The average of ≥9 HFs per mouse quantified from wholemounts is shown (Cre-, n = 4 mice; K14CreEmb^flox/flox^, n = 4 mice; WT, n = 4 mice; *Emb*^*−/−*^, n = 4 mice). (D) Quantification of size of suprabasal sebocytes (WT, n = 72, *Emb*^*−/−*^, n = 72) from Herovici stained sections. Cells were pooled from 3 WT and 3 *Emb*^*−/−*^ mice. Red circles delineate examples of individual suprabasal cells. (E) Quantification of suprabasal sebocyte number per sebaceous gland from cryosections. Average ± SD of mice is shown (2–3-month WT, n = 5; 2–3-month *Emb*^*−/−*^, n = 4; 7-month WT, n = 3; 7-month *Emb*^*−/−*^, n = 3). At least 7 SGs per mouse were quantified. (F) Tail wholemounts from WT and *Emb*^*−/−*^ mice labeled with antibodies against Ki67 (red) and ITGA6 (green) with DAPI nuclear counterstain (blue). (G) Quantification of Ki67+ cells in WT and *Emb*^*−/−*^ SGs (per) SG or normalized to SG size (WT, n = 37; *Emb*^*−/−*^, n = 28). SGs were pooled from 3 WT and 3 *Emb*^*−/−*^ mice. (H) Cryosections of Cre- and K14CreEmb^flox/flox^ mouse tail SG on the same day (0d) or 11 days after (11d) EdU injection stained with antibodies to EdU (green) and basement membrane marker laminin (red) with DAPI nuclear counterstain (blue). (I) Quantification of the percentage of suprabasal EdU+ cells per SG (0-day Cre-, n = 30; 0-day K14 K14CreEmb^flox/flox^, n = 32; 11-day Cre-, n = 48; 11-day K14CreEmb^flox/flox^, n = 45). SGs were pooled from 3 mice per group. (J) Cryosections of neonatal (P1–P2) WT and *Emb*^*−/−*^ tail HFs stained for stearoyl-CoA desaturase-1 (Scd1), which is involved in lipid synthesis (green), and basement membrane marker laminin (red) with DAPI nuclear counterstain (blue). (K) Quantification of SCD1+ cell number per stage V–VI HF (WT, n = 48; *Emb*^*−/−*^, n = 35). HFs were pooled from 4 WT and 3 *Emb*^*−/−*^ mice. In stage V–VI HF, sebocytes can be detected and start forming a sebaceous gland but the SG is not yet localized on the HF posterior wall (Paus et al., 1999). (L) Cryosections of WT and *Emb*^*−/−*^ tail skin stained with a lipid marker LipidTOX (green) and ITGA6 (red) with DAPI nuclear counterstain (blue). (M) Quantification of SG lipids (LipidTOX pixels) in WT and *Emb*^*−/−*^ SGs (WT, n = 30; *Emb*^*−/−*^, n = 18). SGs were pooled from 6 WT and 4 *Emb*^*−/−*^ mice. (N) Quantification of total lipids on mouse skin surface by hexane extraction and TLC (WT, n = 3 mice; *Emb*^*−/−*^, n = 3 mice). Two-tailed t test (C, D, G, I, K, M, and N) or one-tailed t test (E) for independent means was used to determine statistical significance. ^∗∗∗^p < 0.0005, ^∗∗^p < 0.005, ^∗^p < 0.05. The length of scale bars is 50 μm.

To examine the biological function of EMB in the SG, we analyzed the skin of *Emb* knockout mice (Figures 2 and S1C–S1I). Emb^−/−^ mice have increased pre- and peri-natal lethality, but surviving animals have a normal life span (Talvi et al., 2021; Silberstein et al., 2016). The overall appearance of the skin and examination of H&E-stained sections of adult back skin did not reveal any abnormalities in the hair cycle, epidermal thickness, or skin morphology (Figures S1E–S1G). In addition, no change in the numbers of CD45^+^, F4/80^+^, or CD3^+^ immune cells in the epidermis or dermis was detected, suggesting that the epidermal barrier is intact (Figures S1H–S1L) (Cipolat et al., 2014).

While the morphology of the lower HFs was normal in Emb^−/−^ mice (Figures 2A and S1C–S1G), the SGs were significantly larger than those in wild-type (WT) mice. This was also the case in conditional, *K14CreEmb* ^*flox/flox*^, knockout mice, indicating a cell autonomous effect (Figures 2B and 2C). Differentiating SG cells undergo a massive increase in volume in the suprabasal compartment of the SG due to lipid accumulation. However, a detailed examination of WT and Emb^−/−^ SGs showed that neither cell size, measured directly (Figure 2D) or by nuclear density in the suprabasal compartment (Figure S2B), nor the ratio of early- to late-maturing cells determined by LipidTOX staining (Figure S2C) was changed. Thus, the increased size of SGs in *Emb* knockout mice is due to a higher number of sebocytes (Figure 2E). This difference between WT and knockout SGs increased during aging (Figure 2E).

To better understand the underlying reasons for the increased size of *Emb*^*−/−*^ SGs, we employed a heuristic mathematical model that describes the generation of progressively lineage-restricted cells from progenitors (Kirouac et al., 2009). We trained it to describe the approximate cell numbers observed in WT SGs (Figures S2D–S2J and STAR Methods for details). Our model predicted that cell numbers in the SG would be regulated by two key factors: (1) the probability of detachment and differentiation of basal progenitor cells and (2) cell-intrinsic parameters that control the effect of differentiated sebocytes (likely through the size of the SG) on the proliferation rate of basal cells. Comparison of the number of basal progenitor (P) cells and suprabasal differentiating cells (D) provided us with a way to classify the effects of different conditions on SG expansion. The model predicted that expansion of the SG could potentially involve an increase in the number of progenitors (P), the number or size of differentiated cells (D), or a combination of these changes (Figure S2N).

In the case of *Emb*^*−/−*^ mice, we did not detect an accumulation of basal cells (Figures 2H and 2I). SG cells were no more proliferative than WT if normalized to the SG area (Figures 2F and 2G), and the number of EdU+ cells per SG section was not changed (Figure S2P). However, the absolute number of Ki67+ cells per SG was higher in the knockout mice (Figures 2F and 2G), and there was a reduction in cell density in the basal layer (Figure S2O). These observations suggest that *Emb*^*−/−*^ basal progenitor cells tend to move more rapidly into the suprabasal layers of the SG (Figures S2J–S2N), namely, the first of the two factors described by the mathematical model.

To directly measure the movement of basal cells to into the suprabasal SG compartment, we injected adult Cre-negative (Cre-) or K14CreEmb^flox/flox^ mice with EdU and harvested the tissue on the same day or 11 days later. All the EdU+ SG cells were basal at the same day time point (Figures 2H and 2I), and we did not detect a significant difference in the number of EdU+ cells in Emb^−/−^ and WT SGs (Figures 2H, 2I, and S2P). At the 11-day-time-point EdU+ cells were found in the basal and suprabasal layers of knockout and WT SGs, but there was a significantly higher percentage of suprabasal EdU+ cells in K14CreEmb^flox/flox^ SGs (Figures 2H and 2I). We conclude that the phenotype of enlarged SGs in *Emb*^*−/−*^ skin is due to the faster movement of basal SG progenitors into the suprabasal differentiating compartment in adult mice. In contrast, the early morphogenesis of SGs was not affected, based on the quantification of SCD1+ cells in stage V–VI HFs, a time when SGs are starting to form but have not yet localized on the posterior wall of the follicles (Paus et al., 1999) (Figures 2J and 2K).

### EMB regulates monocarboxylate transporters in basal SG cells

Although the sebocyte maturation process in the suprabasal compartment (Figure S2C) and expression of sebocyte markers PPARγ, SCD1, and FASN (Figures S2Q–S2S) were not affected by EMB deletion, the total amount of lipids in *Emb*^*−/−*^ SGs was reduced (Figures 2L and 2M). Based on the hexane extraction of skin surface lipids, there was a slight reduction in sebum production; however, this was not statistically significant (Figure 2N), supporting our macroscopic observation that Emb^−/−^ skin is neither drier nor oilier than control skin. Qualitative analysis of sebum (Figure S2T) did not reveal any differences in abundance of any major lipid classes.

The reduced lipid accumulation in Emb^−/−^ SG could be due to the faster turnover of sebocytes, changes in a rate-limiting lipid synthesis step, or reduced metabolite supply. Interestingly, EMB is known to form a complex with monocarboxylate transporters (MCTs) on the cell surface (Halestrap, 2013). MCTs catalyze the proton-linked transport of monocarboxylates such as L-lactate and pyruvate, and association with EMB or protein family members basigin and neuroplastin is required for correct translocation to the plasma membrane (Halestrap, 2013; Wilson et al., 2005). MCTs are known to transport some precursors and by-products of lipid synthesis (Downie and Kealey, 1998, 2004). Analysis of the scRNA-seq dataset of Joost et al. (Joost et al., 2016) revealed that MCT1 is the only MCT with enriched expression in mouse SGs (Figure S3A). A human scRNA-seq dataset (Cheng et al., 2018) also showed that EMB and MCT1 were co-expressed in human SGs (Figures S3B and S3C).

**Figure 3:**
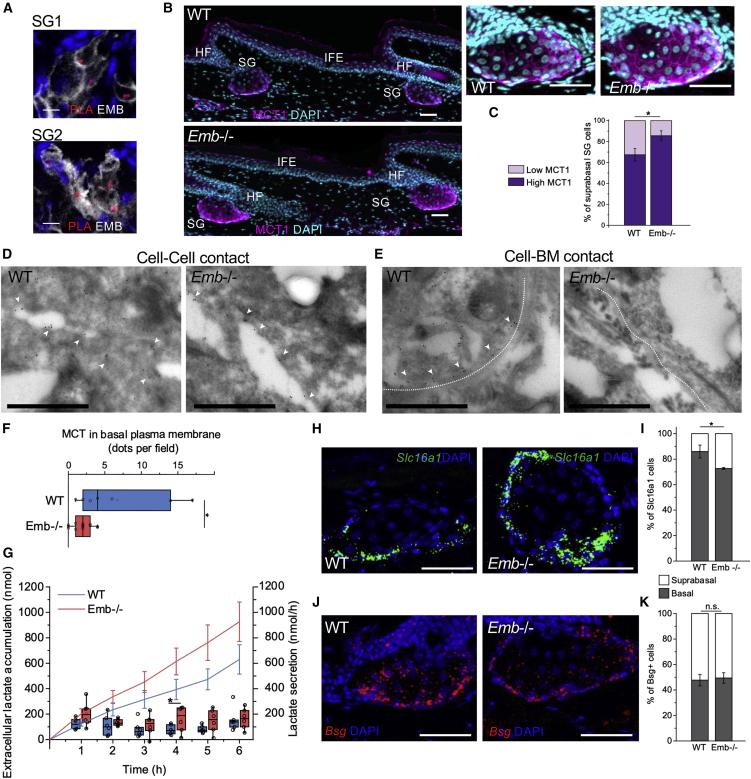
Embigin targets monocarboxylate transport to stromal-progenitor cell interphase (A) Proximity ligation assay between EMB and MCT1 (PLA, red) on mouse tail cryosection stained for EMB (white) and DAPI. (B) Cryosections from WT and *Emb*^*−/−*^ mice stained with MCT1 antibody (magenta) and DAPI nuclear counterstain (blue). (C) Quantification of low MCT1+ versus high MCT1+ cells in suprabasal SG (WT, n = 46; *Emb*^*−/−*^, n = 30), average ± SD is shown. Sebaceous glands were pooled from 3 WT and 3 *Emb*^*−/−*^ mice. (D and E) TEM electron micrographs with MCT1 immunogold labeling showing WT and *Emb*^*−/−*^ basal cell-cell (D) and cell-basement membrane contacts (E). Examples of gold particles in the cell-cell contact or cell-basement membrane (white dotted line) contact are marked with white arrowheads. (F) Quantification of immunogold labeled MCT1 in the basal plasma membrane of basal cells (WT fields, n = 7; *Emb*^*−/−*^ fields, n = 15). TEM electron micrographs were obtained from 2 WT and 2 *Emb*^*−/−*^ mice. (G) Lactate secretion (total nanomoles of lactate, line average ± SEM; speed per hour, box plots) of WT or *Emb*^*−/−*^ tail epidermis as a function of time (WT, n = 6 epidermal pieces from 3 mice; *Emb*^*−/−*^, n = 6 epidermal pieces from 3 mice). (H) Cryosections from WT and *Emb*^*−/−*^ mouse stained with RNAscope against *Slc16a1* (MCT1) (green) and DAPI nuclear counterstain (blue). (I) Quantification of basal/suprabasal % of *Slc16a1* (MCT1)+ cells per SG. Average ± SD of mice is shown (WT, n = 3; *Emb*^*−/−*^, n = 3). At least 8 SGs per mouse were quantified. (J) Cryosections from WT and *Emb*^*−/−*^ mouse skin stained with RNAscope against *Bsg* (basigin) (red) and DAPI nuclear counterstain (blue). (K) Quantification of basal/suprabasal % of *Bsg*+ cells per SG. Average ± SD of mice is shown (WT, n = 3; *Emb*^*−/−*^, n = 3). At least 6 SGs per mouse were quantified. Two-tailed (C, F, I, and K) or one-tailed (G) t test for independent means was used to determine statistical significance. ^∗∗∗^p < 0.0005, ^∗∗^p < 0.005, ^∗^p < 0.05. Scale bars: 50 μm (B, H, and J), 10 μm (A), or 1 μm (D and E).

The co-localization of EMB and MCT1 (Figure S3D) indicated a possible interaction in SGs. To confirm this, we performed a proximity ligation assay (PLA). We detected amplifications in the sebocyte plasma membrane (Figures 3A and S3E), demonstrating that the MCT1-EMB interaction occurs in SGs *in vivo*. Based on confocal images, MCT1 was highly expressed in basal and early suprabasal cells, but MCT1 was reduced and predominantly membrane-associated in more differentiated cells as they moved toward the SD in WT mice (Figures 3B and S3D). However, in *Emb*^*−/−*^ mice, more mature cells in the upper SG still expressed high levels of MCT1 (Figures 3B and 3C). To investigate the subcellular distribution of MCT1 at a higher resolution, immunogold electron microscopical analysis was performed (Figures 3D–3F). In electron micrographs, MCT1 could be detected in the cell-cell contacts of WT and Emb^−/−^ SG cells as well as at the cell-basement-membrane interface of WT SG cells. However, we could not detect the accumulation of MCT1 in cell-basement membrane contacts of Emb^−/−^ SG cells (Figures 3E, 3F, and S3F).

Next, we tested whether *Emb* deletion affected lactate transport *ex vivo* by measuring lactate secretion in epidermal whole mounts. We observed that lactate efflux was increased in *Emb*^*−/−*^ epidermis (Figure 3G), consistent with the high expression of MCT1 in the upper SG (Figure 3C). RNAscope analysis showed that MCT1 was transcribed almost exclusively by basal cells in WT SGs, which explains why protein expression is reduced later in differentiation (Figure 3H). In contrast, in Emb^−/−^ SGs, MCT1 (*Slc16a1*) was also transcribed in suprabasal cells (Figures 3H and 3I).

In summary, the deletion of EMB prevents MCT1 from reaching the basolateral plasma membrane of basal cells. We suggest that this leads to transcriptional compensation and higher expression of MCT1 in suprabasal cells. Given that MCT1 reaches the plasma membrane of suprabasal cells in *Emb*^*−/−*^ SG, it is likely that basigin, which is widely expressed in SGs, compensates for the lack of EMB in maturing sebocytes (Figures 3J and 3K).

### The extracellular domain of EMB is a high-affinity fibronectin receptor

**Figure 4:**
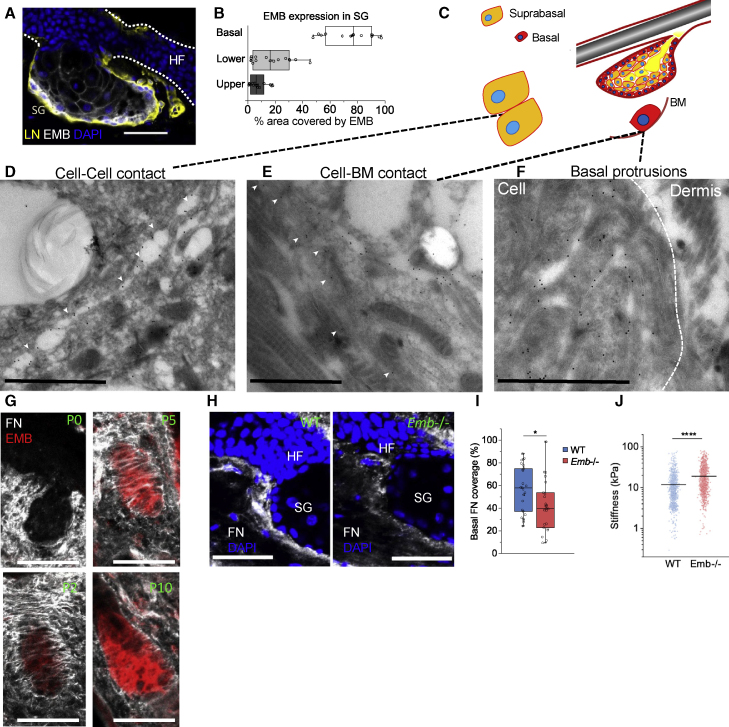
Embigin is concentrated in the basal layer of sebocytes and associates with FN fibers (A) Cryosection of adult SG stained with antibodies to EMB (gray) and a basement membrane marker laminin (LN, yellow) with DAPI nuclear counterstain (blue). (B) Quantification of EMB expression in the different parts of tail sebaceous glands. Box plots show area covered by EMB staining in basal, lower, and upper SG. n = 15 SGs from 5 adult mice. (C) Schematic of SG locations studied by immunogold EM. (D–F) TEM electron micrographs of mouse tail SG with EMB, immunogold labeling showing EMB in suprabasal cell-cell contacts (D), cell-basement membrane contacts (E), and membrane protrusions (F). Gold particles in the cell-cell contact (D) or cell-basement membrane contact (E) are marked with white arrowheads. Dotted line indicates the cell border in (F). (G) Cryosections of mouse back skin collected at P0, P2, P5, and P10 and labeled with antibodies against fibronectin (gray) and EMB (red). (H) Cryosections of adult mouse tail skin stained with antibodies against fibronectin (gray) and counterstained with DAPI (blue). (I) Quantification of proximal fibronectin in peripheral sebaceous glands (WT, n = 25; *Emb*^*−/−*^, n = 23) (Figure S4B). Sebaceous glands were pooled from 5 WT and 4 *Emb*^*−/−*^ mice. (J) AFM stiffness measurements of regions in proximity to the basal SG layer. Individual measurements (WT, n = 1,050; *Emb*^*−/−*^, n = 1,080) were pooled from 4 WT and 4 *Emb*^*−/−*^ mice. Two-tailed t test for independent means was used to determine the statistical significance. ∗∗∗∗p < 0.00005, ∗p < 0.05. Scale bars: 50 μm (A, G, and H) or 1 μm (D–F) in electron micrographs.

The expression of EMB was highest in basal cells, but it could also be detected in the cell-cell contacts of suprabasal sebocytes, except for those that were fully differentiated (Figures 4A and 4B), following the same expression pattern as MCT1 (Figure S3D). By immunogold electron microscopy, EMB was detected *in vivo* on the plasma membrane of sebocyte cell-cell contacts as well on the basolateral plasma membrane of SG progenitor cells (Figures 4C–4F and S4A). A large amount of EMB was also located in the plasma membrane protrusions of basal SG cells (Figure 4F). To examine the possibility that EMB, as a basal cell protein, interacts with the ECM, we co-stained neonatal mouse skin with antibodies against fibronectin (FN) and EMB. EMB co-localized with FN fibers in the developing SGs (Figure 4G). The total amount of FN in skin was not changed in Emb^−/−^ mice (Figure S4C), but basal SG-cell-associated FN was reduced (Figures 4H, 4I, and S4B). No apparent differences were detected in the case of LN expression (Figure S4D) or in hemidesmosome structure and localization (Figures S4E–S4G). However, AFM measurements indicated that tissue stiffness in the proximity of the basal SG was increased upon EMB deletion (Figure 4J), consistent with the known effect of epithelial cell stretching on cell stiffness (Mizutani et al., 2009) and the observed reduction in basal cell density in Emb^−/−^ SGs (Figure S2O).

**Figure 5:**
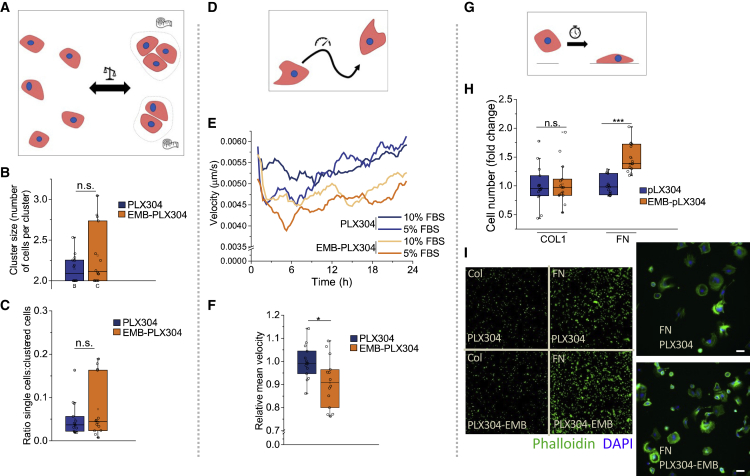
Embigin promotes epithelial cell adhesion to the ECM but does not affect cell-cell adhesion (A–C) Schematic of cell-clustering parameters (A) and cell-clustering analysis (B and C) performed by Operetta (PerkinElmer) (DAPI-phalloidin staining) 2-h time point after seeding cultured mouse keratinocytes overexpressing EMB (EMB-PLX304) or control plasmid (PLX304). The ratios of single cells versus clustered cells (C) and the number of cells in clusters (B) are shown (14 replicates of each condition from 3 independent experiments normalized to the average of controls in each experiment). (D–F) Schematic (D) and analysis of random migration (Livecyte, Phase Focus Limited) in EMB-overexpressing and control keratinocytes. (E) The 2-h average velocity trendline (2 replicates in each condition) (E) and (F) 24-h average velocity (14 replicates of each condition from 3 independent experiments normalized to the average of controls in each experiment) (F) are shown. (G and H) Schematic (G) and cell adhesion analysis of plate-and-wash assay comparing EMB-overexpressing and control keratinocytes 2 h after seeding on collagen or fibronectin (H). Cell number was quantified by Operetta (DAPI-positive nucleus). 15 replicates of each condition from 3 independent experiments normalized to the average of controls in each experiment are shown. (I) Representative images of the 2-h plate-and-wash assay stained with phalloidin (green) and DAPI (blue). Two-tailed t test for independent means was used to determine the statistical significance. ^∗∗∗^p < 0.0005, ^∗∗^p < 0.005, ^∗^p < 0.05. Scale bars: 50 μm.

Given the localization of EMB at the basement zone of SG basal cells as well as its role in promoting the detachment of basal progenitor cells (Figure 2) and in the basal targeting of MCT1 (Figure 3), we hypothesized that the primary role of EMB is to modulate the interaction between cells and the ECM. To test this, we compared spontaneously immortalized mouse epidermal cells overexpressing EMB with vector control cells. The analysis of cell-cell adhesion by high-content imaging (Operetta, PerkinElmer) did not show any effect on the ratio of single cells versus clustered cells or on the number of cells in the clusters between the control and EMB-overexpressing cells (Figures 5A–5C and S5A). In contrast, migration analysis using quantitative phase imaging (Livecyte, Phase Focus) revealed that the overexpression of EMB reduced the instantaneous migration velocity (Figures 5D–5F). In addition, plate-and-wash assays showed higher attachment of EMB-overexpressing cells to FN-coated wells (Figures 5G–5I). The role of EMB in cell adhesion to FN was confirmed by using xCELLigence RTCA (ACEA Biosciences) impedance-based technology: the siRNA-mediated knockdown of EMB decreased and the overexpression of EMB increased cell adhesion to FN (Figures S5B–S5E). In contrast to its effect on FN adhesion, EMB overexpression did not increase the attachment to collagen (COL)-coated wells (Figure 5H). EMB had only a small impact on the morphological parameters of the cells, supporting the idea that the primary role of EMB is to increase the strength of the interaction between the ECM and cells in the attachment (Figures 5I and S5F).

**Figure 6:**
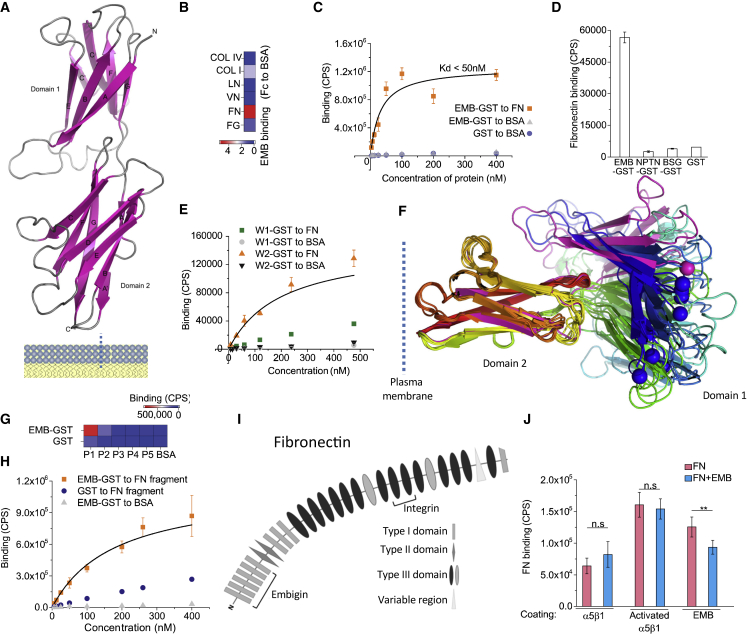
Fibronectin binds EMB via a flexible plasma-membrane-distant immunoglobulin domain of EMB and type I fibronectin repeats (A) 3D model of embigin extracellular domains. (B) Binding of recombinant EMB-GST (200 nM) to different ECM ligands as fold change relative to BSA control. Averages of 3 replicates are shown. (C) Binding of recombinant EMB-GST to fibronectin and controls as a function of concentration. Averages of 3 replicates ± SD are shown. (D) Binding of recombinant EMB-GST, BSG-GST, NPTN-GST, and GST to fibronectin. Averages of 3 replicates ± SD are shown. (E) Binding of recombinant EMBW1-GST (no Ig1 domain) and EMBW2-GST (no Ig2 domain) proteins to fibronectin and controls as a function of concentration. Averages of 3 replicates ± SD are shown. (F) Flexibility of the EMB extracellular domain. The angle between domain 1 and domain two differs in all the 9 models created by Modeller using the default parameters. The fold is colored with rainbow colors from the blue N terminus to the red C terminus. The representative model is colored in magenta. The alpha-C carbon atoms of the N-terminal residues are shown as spheres to depict the difference in the 3D position of the N terminus in each model. (G) Binding of recombinant EMB-GST (200 nM) to different fibronectin fragments (starting from N terminus toward C terminus: P1, N-terminal heparin-binding fragment; P2, 45-kDa gelatin-binding fragment; P3, Ser607-Pro1265; P4, Glu1266-Pro1908; P5, Val1913-Glu2477). Averages of 3 replicates are shown. (H) Binding of recombinant EMB-GST to N-terminal 30-kDa fragment (P1) and controls as a function of concentration. Averages of 3 replicates ± SD are shown. (I) An illustration of the FN domain structure as well as RGD, major integrin binding site, and EMB. (J) Fibronectin (10 μg/mL) binding to recombinant extracellular domain of integrin α5β1 or EMB-GST in the presence or absence of soluble EMB-GST (10 μg/mL). Averages of 6 replicates ± SD are shown. Two-tailed t test for independent means was used for determining the statistical significance. ^∗∗∗^p < 0.0005, ^∗∗^p < 0.005, ^∗^p < 0.05.

To examine the mechanism by which EMB promoted cell adhesion to FN, we focused on structural-functional studies of the EMB core protein. We created a 3D homology model of the EMB extracellular domain and produced it as a GST-fusion protein (Figures 6A, S6A, and S6B). In solid-phase binding assays, EMB bound to FN but not to COL I, COL IV, vitronectin, or LN (Figures 6B and S6C), consistent with the results of our cultured cell adhesion assays (Figure 5H). The binding of EMB to FN was a high-affinity (Figure 6C), metal-ion-independent (Figure S6D) interaction. The recombinant extracellular domains of basigin and neuroplastin did not bind to FN in our assays (Figure 6D), indicating that the FN interaction is a unique feature of EMB within its protein family.

To map which domain EMB uses in binding to FN, we separately deleted the immunoglobulin folds from the recombinant protein. Deletion of the Ig2 fold, the Ig domain closer to the plasma membrane, had very little effect on binding, whereas deletion of the distant Ig1 domain inhibited the binding of EMB to FN (Figure 6E). The structural comparison of EMB, basigin, and neuroplastin revealed that Ig1 is less conserved than the Ig2 domain, supporting our finding that neither basigin nor neuroplastin interact with FN (Figures S6E and S6F). Interestingly, structural modeling indicates a high flexibility between the Ig1 and Ig2 domains because each of the 9 EMB models created by Modeller (Sali and Blundell, 1993) using default parameters adopts a different angle between these two domains (Figure 6F). Given our binding results, the vast flexibility of the Ig2 domain would likely increase the capability of EMB to bind to large immobile ligands such as FN.

Next, we identified the EMB binding site in FN. By using FN fragments, we found that the EMB ectodomain binds to the N-terminal 30-kDa fragment of FN, which is formed of type I repeats (Figures 6G and 6H). In contrast, the major integrin binding site, containing the RGD motif, is located in the type III FN domain (Maurer et al., 2015; Ruoslahti, 1996) (Figure 6I). Consistent with this, recombinant heterodimeric α5β1 integrin and the EMB ectodomain could simultaneously bind to FN in a competition assay (Figure 6J).

In summary, FN is a direct high-affinity ligand for EMB that is able to mediate cell adhesion in addition to integrins.

## Discussion

Our results uncover EMB as playing two major roles in basal SG cells. First, EMB regulates the expression and function of MCT1 in basal SG cells, permeabilizing the cells to metabolite flow. Secondly, EMB increases the adhesion of basal SG cells to the ECM. Based on biochemical and cell biological analysis, we have provided a detailed, previously unknown, mechanism explaining how EMB regulates cell adhesion: the outer extracellular domain of EMB binds to the N-terminal type I domains of FN. MCTs interact with EMB, basigin, and neuroplastin via a partially similar mechanism, although specific MCTs interact preferentially with certain ancillary proteins (Halestrap, 2013). Notably, only EMB binds to FN (Figure 6), suggesting that the selectivity of extracellular ligands could be a key factor that led to the divergent evolution as well as tissue-specific expression patterns of this protein family.

In dermal-epidermal junctions, FN is localized in the lamina lucida area of the basement membrane and in proximity to the plasma membrane of basal epidermal cells (Couchman et al., 1979; Fleischmajer and Timpl, 1984), indicating that FN is a true physiological ligand for EMB. FN surrounding the SG is intensively modified during SG development. The transition from the expansion to the homeostatic phase in developing SGs has recently been shown to correlate with stromal changes that affect the biomechanical properties of the niche (Andersen et al., 2019). Our studies show that it is likely that FN is one of the stromal factors involved.

Our results suggest that the attachment of basal progenitor cells to FN is an important feature of the sebocyte homeostatic program since EMB knockout mice have altered SG homeostasis, biased toward the detachment of progenitor cells. As a result, there is a higher number of differentiating cells in adult SGs of EMB knockout versus WT mice (Figure 2). The model whereby EMB drives the transition from the progenitor compartment to differentiation by regulating cell adhesion provides a possible mechanism of EMB function in other tissues as well (Guenette et al., 1997; Silberstein et al., 2016). For instance, inhibiting EMB in the bone marrow with a function-blocking antibody leads to the mobilization of certain stem cell and progenitor cell populations (Silberstein et al., 2016), supporting the idea of an adhesive function of EMB in hematopoietic stem cells.

The role of integrin-mediated adhesion in regulating epidermal stem cell proliferation and differentiation is well established (Adams and Watt, 1989; Watt and Fujiwara, 2011). Our results demonstrate that EMB functions independently of integrins at the molecular level by binding to a region of FN that is distant from the RGD motif, the major binding site of integrins. Notably, EMB does not compete with integrins for FN binding, which is in line with the previous hypothesis that EMB enhances integrin-mediated adhesion to the ECM (Huang et al., 1993). An interesting curiosity is that the N-terminal 30-kDa domain of FN, where the EMB interaction site lies, is required for FN fibril assembly. The involvement of any cellular receptors in 30-kDa binding has remained elusive (Lemmon and Weinberg, 2017; Singh et al., 2010) until now. The role of EMB as a niche-interacting factor as well as the altered FN staining in EMB knockout SGs (Figure 4) does not preclude the possibility that EMB itself can participate in FN fibril assembly. However, this hypothesis would require further investigation.

The understanding of niche interactions and their molecular characterization have not only provided insights into how adult stem cell renewal and differentiation are maintained in tissues but have also suggested direct translational potential. One example of this is the use of specific niche ECM components such as LN-511 for the *in vitro* culture of epidermal stem cells for human skin grafting (Tjin et al., 2018). Another example is the use of soluble factors, such as angiogenin secreted by the hematopoietic niche, to support the grafting of bone marrow stem cells (Goncalves et al., 2016). In our studies, the ability of EMB to modulate lipid production of SGs without affecting overall skin homeostasis suggests that the blocking of EMB could be an attractive therapy for skin conditions associated with excessive production of lipids, such as acne vulgaris. Further studies building on our discovery will likely widen the translational potential of our findings to other tissues and to cancer. In summary, our results identify a factor regulating the interaction between SG progenitor cells and their niche and highlight the need to understand the contribution of less-well-studied proteins to stem cell function.

### Limitations of the study

The short-term consequences of EMB depletion in adult mice were not studied, since EMB is deleted in Emb^−/−^ or K14Cre Emb^flox/flox^ mice before SG development. Our lipid analysis cannot rule out the possibility of changes in single lipid classes due to Emb depletion. We did not study whether EMB knockout affects other monocarboxylate transporters in the same way as MCT1.

## STAR★Methods

### Key resources table

**Table:** 

REAGENT or RESOURCE	SOURCE	IDENTIFIER
**Antibodies**		

CD3	Abcam	SP7, ab16669; RRID: AB_443425
CD45	Biopharmingen	Clone 30-F11, #553076; RRID: AB_394606
EMB	In-house (Stephen L. Nutt), Pridans et al., 2008	G7.43.1
F4/80	BioLegend	BM8, #123115; RRID: AB_893493
FASN	Santa Cruz Biotechnology	G-11, sc48357; RRID: AB_627584
FN	Abcam	ab2413; RRID: AB_2262874
GATA6	Cell Signalling	D61 E4 clone, #5851; RRID: AB_10705521
GST (Delfia Eu-N1-anti-GST)	PerkinElmer	AD0251
ITGα6-FITC	eBioscience	GoH3 clone, 14-0495-82; RRID: AB_891480
K14	BioLegend	Poly19053; #905303; RRID: AB_2734678
Ki67	Abcam	SP6, ab16667; RRID: AB_302459
Laminin	Sigma	L9393; RRID: AB_477163
LRIG1	R&D Biosystems	#AF3688; RRID: AB_2138836
MCT1	Millipore	AB1286-1; RRID: AB_90565
PPARγ	Santa Cruz Biotechnology	E-8, sc7273; RRID: AB_628115
SCD1	Cell Signaling	C12H5, #2794; RRID: AB_2183099
SCD1	R&D Biosystems	Clone#428208, #MAB4404; RRID: AB_2183118

**Bacterial and virus strains**		

BL21Tuner	Novagen (Sigma- Aldrich)	70622-M
BL21Tuner-pGex-2T-Embigin ecto	This paper	N/A
BL21Tuner-pGex-2T-Neuroplastin ecto	This paper	N/A
BL21Tuner-pGex-2T-Basigin ecto	This paper	N/A
BL21Tuner-pGex-2T-W1Embigin	This paper	N/A
BL21Tuner-pGex-2T-W2Embigin	This paper	N/A

**Chemicals, peptides, and recombinant proteins**		

Fibronectin N-terminal heparin binding fragment	Sigma-Aldrich	F9911
Fibronectin 45kDa gelatin binding fragment	Sigma-Aldrich	F0162
Recombinant Human Fibronectin Fragment 2 (Ser607-Pro1265),	R&D Systems	#3225-FN
Recombinant Human Fibronectin Fragment 3 (Glu1266-Pro1908)	R&D Systems	#3938-FN
Recombinant Human Fibronectin Fragment 4 (Val1913-Glu2477)	R&D Systems	#3624-FN
Corning™ Fibronectin, Human	Thermo Fisher Scientific	11533610
Recombinant Human Integrin alpha5beta1	R&D Systems	#3230-A5-050
ProLong Gold Antifade Mountant	ThermoFisher, Canoga Park, California	ref: P36930
5-Ethynyl-2′-deoxyuridine	Sigma	900584

**Critical commercial assays**		

Protino Glutathione Agarose 4B	Macherey-Nagel Germany	REF 745500.100
RNAscope Multiplex Fluorescent Detection Kit v2	ACDBio, Newark, California	ref: 323100
RNAscope Slc16a1 (mouse)	ACDBio, Newark, California	ref: 423661-C2
RNAscope Bsg (mouse)	ACDBio, Newark, California	ref: 491721)
Opal 570 Reagent Pack dye	Akoya Biosciences, Marlborough, Massachusetts	ref: FP1488001KT
L-lactate assay kit	Abcam	ab65330
Duolink In Situ Wash Buffers, Fluorescence	Sigma	DUO82049
Duolink In Situ Mounting Medium with DAPI	Sigma	DUO82040
Duolink In Situ Probemaker PLUS	Sigma	DUO92009-1KT
Duolink In Situ Probemaker MINUS	Sigma	DUO92010-1KT
Duolink In Situ Detection Reagents Red	Sigma	DUO92008-30RXN
Silica gel on TLC plates, L × W 20 cm × 20 cm, fluorescent indicator: no,	Merck	99570-25EA
Methyl cis-15-tetracosenoate	Merck	17265
Click-iT™ EdU Imaging Kit with Alexa Fluor™ 488	Invitrogen	C10337

**Deposited data**		

Analysis of published mouse skin scRNA-seq data	Joost et al., 2016	GEO: GSE67602
Analysis of published human skin scRNA-seq data	Cheng et al., 2018	EGA: EGAS00001002927
Basigin crystal structure	Wright et al., 2014	PDB: 4U0Q
Basigin crystal structure	Yu et al., 2008	PDB: 3BH5
Neuroplastin crystal structure	Owczarek et al., 2010	PDB: 2WV3
Neuroplastin crystal structure	Gong et al., 2018	PDB: 6A69

**Experimental models: cell lines**		

Mouse skin keratinocytes	In-house, Ghahramani et al. 2018	N/A

**Experimental models: organisms/strains**		

Mouse: K14ΔNLef1	In-house, Nieman et al., 2002	N/A
Mouse: K14ΔNβ-CateninER (D2 line)	In-house, Lo Celso et al., 2004	N/A
Mouse: TopH2BeGFP	Ferrer-Vaquer et al., 2010	N/A
Mouse: TUKO53 Emb-/-	In-house, Silberstein et al., 2016	N/A
Mouse: K14Cre	Dassule et al., 2000	N/A
Mouse Emb *flox/flox*	Talvi et al., 2021	N/A

**Oligonucleotides**		

Mm_Emb_1 Flexitube siRNA	QIAGEN	Cat#SI00993307
Mm_Emb_4 Flexitube siRNA	QIAGEN	Cat#SI00993328
AllStars Negative Control siRNA	QIAGEN	Cat#1027280
RNAscope mouse Bsg	ACDBio	ref: 491721
RNAscope mouse Slc16a1	ACDBio	ref: 423661-C2

**Recombinant DNA**		

CCSB-Broad LentiORF - EMB Clone Accession: BC059398 Clone ID: ccsbBroad304_04883 glycerol stock	Horizon Discovery Ltd (Dharmacon)	OHS6085-213577454
PLX304	Adgene	#25890
pcDNA3.1+-EMB	This paper	N/A
pGex-2T-Embigin ecto	This paper	N/A
pGex-2T-Neuroplastin ecto	This paper	N/A
pGex-2T-Basigin ecto	This paper	N/A
pGex-2T-W1Embigin	This paper	N/A
pGex-2T-W2Embigin	This paper	N/A

**Software and algorithms**		

Origin 2016	OriginLab Corporation	N/A
GraphPad Prism 6	GraphPad Software	RRID:SCR_00279
Harmony 4.8 High-Content Imaging and Analysis Software	PerkinElmer	HH17000001
Phasefocus Analyse V3.0.1 + Random Motility Dashboard	Phase Focus Limited	N/A
NDP.view2 (U12388-01)	Hamahatsu	N/A
Fiji – ImageJ	Schindelin et al., 2012	N/A

### Resource availability

#### Lead contact

Lead Contact, Professor Fiona Watt (fiona.watt@kcl.ac.uk).

#### Materials availability

All unique/stable reagents generated in this study are available from the lead contact with a completed Materials Transfer Agreement.

### Experimental model and subject details

#### Mouse models

The mice lines used for this study included K14ΔNLef1 (Niemann et al., 2002), K14ΔNβ-CateninER (D2 line) (Lo Celso et al., 2004), TopH2BeGFP (Ferrer-Vaquer et al., 2010), K14Cre (Tg(KRT14-cre)1Amc MGI, Dassule et al., 2000), TUKO53 Emb-/- and Emb^flox/flox^ (Talvi et al., 2021; Silberstein et al., 2016). All mice used in the experiments were on C57BL6/CBA or mixed backgrounds. Comparison between wildtype and knockout animals was performed by using F1 littermates. Both sexes were used in the analysis. In the case of K14ΔNβ-CateninER mice, the transgene was activated by five topical applications of 4-hydroxytamoxifen (1.5mg in acetone, Sigma Aldrich). To study basal cell detachment (Weinstein, 1974), mice were treated with EdU (intraperitoneal injection, 5-Ethynyl-2'-deoxyuridine, 5mg/ml in PBS, 200μl per mouse, Invitrogen) and culled 4-6h or 11 days later. All animal work was approved locally at University of Turku (Finland) and King’s College London (UK) and performed with Finnish Ethical Committee approval or under a UK Government Home Office license (PPL 70/8474).

#### Mouse keratinocyte culture

Mouse keratinocytes, isolated from adult dorsal skin (Carroll et al., 1995), were cultured in FAD medium (Watt et al., 2006) comprising three parts DMEM medium (Lonza), one part Ham’s F12 medium (Thermo Fisher Scientific) supplemented with 10% FCS (Biowest), 2 mM L-glutamine (Lonza), 10 units/ml Pen-Strep (Lonza), 200 μM adenine (Sigma-Aldrich), 0.5μg/ml hydrocortisone (Sigma-Aldrich), 5μg/ml insulin (Sigma-Aldrich), 16,8 ng/ml cholera toxin (Sigma-Aldrich) and 10 ng/ml epidermal growth factor (Sigma-Aldrich). To overexpress EMB, subconfluent mouse keratinocytes were transfected with human EMB cDNA in PLX304 vector (CCSB-Broad LentiORF - EMB Clone Accession: BC059398 Clone ID: ccsbBroad304_04883 glycerol stock, Dharmacon) by using lentiviral particles in culture medium containing 5 μg ml^−1^ polybrene (EMD Millipore), as described earlier (Oulès et al., 2020). Cells were detached by accutase (Biolegend). siRNA mediated silencing of EMB in mouse keratinocytes (Mm_Emb_1, Mm_Emb_4 Flexitube siRNAs or AllStars Negative Control siRNA from QIAGEN) was performed using siLentFect reagent (Bio-Rad) according to the manufacturer’s instructions. Cells were used for experiments within 24h after transfection. For overexpression of EMB, keratinocytes were transfected with pcDNA3.1+-EMB or the vector alone by using HilyMax (Dojindo) according to the manufacturer’s instructions. After 24 h, Emb positive cells were selected and maintained in medium containing 0.5 mg/ml of G418 (Invitrogen). The efficiency of transfection was tested by Western blotting with anti-EMB antibody (clone G7.43.1, eBioscience) and anti-β-Tubulin I monoclonal antibody (Clone: SAP.4G, T7816, Sigma-Aldrich) as a control.

### Method details

#### Histology and antibody staining

Antibody staining was performed on OCT embedded skin (12μm sections) or tail epidermal whole mounts as described previously (Liakath-Ali et al., 2014; Philippeos et al., 2018). The following primary antibodies (dilutions in brackets) were used: CD3 (1:100, SP7, Abcam), CD45 (1:500, Clone 30-F11, Biopharmingen), EMB (1:1000, G7.43.1 (Pridans et al., 2008)), F4/80 (1:250, BM8, BioLegend), FASN (1:100, G11, Santa Cruz), FN (1:250, Ab2413, Abcam) GATA6 (1:200, D61E4 clone, Cell Signalling 5851), ITGα6-FITC (1:200, GoH3 clone, eBioscience 14-0495-82), K14 (1:500, Poly19053, BioLegend) Ki67 (1:500, SP6, Abcam), Pan laminin (1:1000, Sigma L9393), LRIG1 (1:250, R&D Biosystems AF3688), MCT1 (1:1000, AB1286-1, Millipore), PPARγ (1:100, E-8, Santa Cruz), and SCD1 (1:250, C12H5, Cell Signaling; 1:250, MAB4404, R&D Biosystems). The samples were stained with Alexa fluor -conjugated secondary antibodies (1:500, ThermoFisher Scientific). In some cases, LipidTOX HCS LipidTOX Deep Red Neutral lipid stain (1:500, Life Technologies) and DAPI (1ng/ml) were incubated simultaneously with secondary antibodies. EdU was stained with a Click-iT EdU Alexa Fluor 488 imaging Kit (Invitrogen) by following the manufacturer’s protocol. In addition, some sections were stained with hematoxylin & eosin or Herovici. Images were acquired with a Nikon A1 confocal microscope or NanoZoomer Slide Scanner (Hamamatsu). Images were analyzed (cell counting, width, length, thresholding pixels) using Fiji ImageJ.

#### RNAscope multiplex fluorescent assay

12 μm skin sections from cryoblocks were fixed in 4% PFA then analyzed by RNA hybridization using the RNAscope Multiplex Fluorescent Detection Kit v2 (ACDBio, Newark, California, ref: 323100), following the manufacturer’s instructions. Probes against mouse Slc16a1 (ACDBio, ref: 423661-C2) and Bsg (ACDBio, ref: 491721) mRNA molecules were used. Opal 570 Reagent Pack dye (Akoya Biosciences, Marlborough, Massachusetts, ref: FP1488001KT) was used at a dilution of 1:1,000 at the fluorophore step to develop *in-situ* hybridised probes. Nuclei were counterstained with 4',6-diamidino- 2-phenylindole (DAPI) and mounted using ProLong Gold Antifade Mountant (ThermoFisher, Canoga Park, California, ref: P36930). The experiment was performed on three skin samples each from a different mouse for each condition (control or *Emb-/-*, six mice in total). Slides were imaged with a Nikon A1 confocal microscope and the number of positive cells was counted manually using Fiji ImageJ.

#### Proximity ligation assay (PLA)

Before the assay, PLUS and MINUS probes were prepared by direct crosslinking to EMB (G7.43.1) and MCT1 (AB1286-1, Millipore) antibodies in PBS using the Duolink In Situ Probemaker kit (Merck). PLA was performed on 12 μm sections of OCT embedded mouse tail skin fixed in 4% PFA (10min, RT). Blocking, probe incubation (1:100, o/n, +4°C), ligation, and amplification were performed with the Duolink In Situ Detection (Red) kit (Merck). Secondary antibody against rat IgG (1:500 in PBS, ThermoFisher Scientific) was added after finishing the PLA (1h, RT). After washing away the secondary antibody (3xPBS, 10min), the samples were mounted in Duolink In Situ Mounting Medium with DAPI. Slides were imaged with a Nikon A1 confocal microscope.

#### Sebum analysis

Sebum was analyzed by using thin layer chromatography as described previously (Greene et al., 1970). 4ml of hexane with methylmervonate (50μg/ml, Merck) was applied to harvested mouse back skin (with hairs) in a polybrene cylinder (5cm^2^). After 45s, 3ml of the hexane extract was dried under N_2_ flow and resuspended in 150μl of hexane. A human sebum sample was collected by touching skin with a pipet tip followed by hexane extraction from the pipet tip. 10μl samples were applied to an activated TLC plate (30min, 100°C, Silica gel on TLC plates, 20cm x 20cm, Merck). TLC was developed in a TLC tank with three solvents: 1) hexane up to 19cm, 2) benzene up to 19cm, 3 hexane : diethyl ether : glacial acetic acid (70:30:1) up to 10cm. The plate was dried between solvent extractions. Lipids in the TLC plate were visualized by spraying the plate with 50% sulfuric acid and heating (200°C) until bands appeared. The cooled down plates were imaged and quantified using Fiji ImageJ and an internal standard (methylmervonate).The bands were recognized based on their published retention in TLC (Greene et al., 1970).

#### Lactate export assay

Tail epidermal sheets were detached by incubating (30min, 37°C) tail skin (0.6cm x 0.6cm) in Dispase solution (1:5 dilution in PBS, 50 caseinolytic units per ml, Corning) and carefully peeling. Epidermal sheets were washed (3x5min) in ice cold Krebs-Henseleit buffer (118mM NaCl, 4.7mM KCl, 1.2mM MgCl_2_, 1.25mM CaCl_2_, 1.2 KH_2_PO_4_, 25mM NaHCO_3_, 11mM D-glucose). After washing, the epidermal sheets were incubated in the same buffer for up to 6 hours (37°C) and samples were collected once per hour. The lactate concentration of the samples was determined with a colorimetric L-lactate assay kit (Abcam).

#### Data analysis

The published list of marker genes (Joost et al., 2016), based on the first level clustering of single cell RNA-seq data, was filtered with GO terms for cell-cell adhesion mediator (GO:0098632) and cell-matrix adhesion (GO:0007160), and ranked based on the fold change of expression (compartment vs. baseline).

#### Mathematical modelling of cell numbers in developing sebaceous glands

As shown in the main text, we observed a significant difference between the cell numbers (both basal, and suprabasal) in the sebaceous glands (SGs) of wildtype (WT) and Embigin knockout (*Emb*^*-/-*^) mice (Figures 2 and S2). To understand the possible underlying drivers that might control the total cell numbers in a fully developed SG, we opted to develop a simplified mathematical model that described the development of the committed SG progenitors into a fully developed SG and query it for insight.

We chose the simplified approach of stem-cell-derived tissue development that was reported by Kirouac et al. (Kirouac et al., 2009) for the hematopoietic system and adapted it to the development of the SG. In their model, Kirouac et al. considered a tissue comprised of multiple cell compartments along the stem cell hierarchy. The first compartment represented the tissue progenitors, and all the following compartments represented progressively more committed lineages until the final compartment represented the terminally differentiated cell type (Figure S2D) that was unable to self-renew. Each compartment in such a model is described by two characteristic functions. First, its growth rate $u_{P}$, and second, its probability of self-renewal $f_{P}$ (Figure S2E). In this model, the rate change in cell numbers for any compartment can be described by the expression shown in Figure S2F. Given that the SG is comprised of two cell types, the basal (‘P’) and the suprabasal cells (‘D’), and that the progenitor (basal) cells are the only proliferating cell compartment, the rate change of the P and D compartments simplify to the following:

$$\frac{dP}{dt}=\left(2f_{P}-1\right)\;u_{P\;}P$$

$$\frac{dD}{dt}=\left(1-\;f_{P}\right)\;u_{P}\;P$$

As a first step toward deriving a complete mathematical description of our system, we elected to further describe the growth rate. In a recent study that followed the development of SGs in the back skin of mice, the authors observed that the development of SGs started at around P2 with a pool of 11 progenitors and ceased at around P7 (Andersen et al., 2019). In line with their observations, we opted to employ a Gaussian decay function to describe the growth rate of the progenitor compartment. Notably, whereas Andersen et al. studied developing SGs in the back skin, our data is from tail skin SGs. Andersen et al. found that back skin SGs were comprised of ∼30 cells at the end of the development period. The cell numbers in tail skin SGs during homeostasis are much higher – ∼250 cells in WT mice – as seen estimated by our data (Figures S2A, S2B, and S2O; see quantification and statistical analysis). This could happen through differences in three parameters during development: first, the starting number of progenitors; second, the rate at which basal cells in tail skin SGs proliferate; and third, the duration of SG development in tail skin. Given that an increase in the cell cycle rate of basal cells in tail skin SGs is unlikely, we discounted the possibility of a different cell cycle rate accounting for the differences in the numbers of SG cells in tail skin. For the sake of simplicity, we assumed that SGs in tail skin start from a pool of 11 progenitors – similar to back skin, but that the development of tail skin SGs proceeds for longer than that of back skin SGs. Empirically, we found that a development till P9 described our observations well. While these assumptions fit our observations well, further work is required to test these in more detail.

As the basal cells in the developing SG lift off and undergo the differentiation program, they dramatically increase in size. The rapid increase in SG volume caused by cells lifting off the basement membrane, further amplified by the increasing size of differentiated cells, would increase the surface area of the basement membrane and cause basal cells to proliferate in order to maintain the physical integrity of the basal layer. Given this, we incorporated a feedback from the ratio between the suprabasal cells to basal cells ($\frac{D}{P}$) to the growth rate. Overall, the growth rate took the form:

$$u_{P}=c\;{\left(1+\;\frac{D}{P}\right)}^{k}\;e^{-t^{2}}\;$$

c,k→constants

Overall, a complete mathematical description of the development of tail skin SGs can be simplified to the equations show in Figure S2I. We trained the unknown parameters of these equations based on the cell numbers that we see in the WT SGs. The final parameter values are shown in Table S1.

#### Electron microscopy

Immuno electron microscopy was performed at the Biocenter Oulu Electron Microscopy Core Facility. Fresh skin samples from adult mouse tail were fixed with paraformaldehyde (4% in 0.1 M phosphate buffer with 2.5 % sucrose, pH 7.4). After fixation, the samples were immersed in sucrose (2.3M), frozen in liquid nitrogen, and sectioned with a Leica EM UC7 cryoultramicrotome (Leica Microsystems, Vienna, Austria). For immunolabeling, sections on Butvar-coated nickel grids were first incubated in 0.1% glycine-PBS for 10 min followed by blocking (serum, 1% BSA in PBS for 5 min). Primary antibodies, anti-EMB (G7.43.1) and anti-MCT1 (AB1286-1, Millipore), were incubated for 45 min and corresponding secondaries, anti-Rat and anti-Chicken IgG (Jackson Immunoresearch Laboratories Inc. Baltimore, PA, USA), for 30 min followed by incubation with protein A conjugated 10 nm gold (Cell Microscopy Core, University Medical Center Utrecht, The Netherlands) for 30 min. 1% BSA in PBS was used in washing steps and dilutions of antibodies and gold conjugates. The grids were stained with neutral uranyl acetate (UA) and coated with 2% methyl cellulose with 0.4 % UA. Tecnai G2 Spirit 120 kV transmission electron microscope (FEI, Eindhoven, The Netherlands) was used for imaging and Quemesa CCD camera (Olympus Soft Imaging Solutions GMBH, Münster, Germany) for capturing the images.

#### Sebaceous gland AFM measurements

The AFM measurements were carried out using a Bioscope atomic force microscope (Bioscope resolve™ BioAFM, Bruker), coupled with an optical microscope (Leica). Fresh-frozen mouse skin was sectioned at 10 μm thickness and sections were attached to superfrost™ microscope slides. Prior to measurements, the sections were washed with phosphate buffered saline (PBS) three times to remove residual optical cutting temperature compound (OCT). 3 sebaceous glands per skin section were chosen at random, and for each gland 3 areas of 5 x 5 μm were selected. On average 30 force-curves were included for each area. The results presented are based on approximately 1000 individual measurements pooled from 4 animals per group (WT and KO). A spherical nitride tip (5μm) on a nitride lever (SAA-SPH-5UM, Bruker) was used. The Poisson’s ratio was 0.5mm, and the minimum force fit boundary and maximum force fit boundary were 30% and 70% respectively. Trigger force was 10nN and the ramp size was 10nm. The Young’s modulus was calculated by fitting the force-curves with modulus fit model Hertzian (spherical) using the Nanoscope analysis software 1.8 (Bruker).

#### Adhesion experiments

For cell adhesion and migration assays, tissue culture plates were coated with collagen (5mg/cm^2^, collagen I from rat tail tendon in 0.02N acetic acid) or (5mg/cm^2^, Corning) in PBS overnight at +4°C or 1h at +37°C. Serum was not used in adhesion assays. Cell-cell clustering and plate-and-wash assays were extensively washed with PBS two hours after seeding 10 000 cells per well in 96-well plates. The cells were fixed (10min, 4% PFA) and stained with DAPI and Phalloidin-488 (Fisher Scientific). Images were acquired with the PerkinElmer Operetta CLS High-Content Imaging System and analyzed using custom algorithms in the PerkinElmer Harmony high-content analysis software package (Figure S5A; STAR Methods). Migration was assayed with a Phasefocus LivecyteTM (Phase Focus Limited) on sub confluent 96-well plates. For the real time cell adhesion experiment, cells were detached with 5 mM EDTA and the adhesion of 15 000 cells to fibronectin (5 mg/cm2 in PBS, o/n, +4°C, Sigma-Aldrich) or BSA (1 mg/ml in PBS, o/n) coated wells was measured by xCELLigence RTCA (ACEA Biosciences Inc.).

#### Cell cluster analysis by high-content image analysis

Images obtained by Operetta CLS (PerkinElmer) were analyzed with the Harmony software package (Figure S5A). Nuclei were initially defined using the DAPI channel. Small (< 85 μm^2^), exceedingly large (> 1000 μm^2^) and highly irregular (roundness < 0.65) nuclei were excluded from the analysis. The cytoplasm of cells was subsequently marked based on phalloidin-mediated actin staining. A sliding parabola filter was applied to phalloidin stained images to remove imaging artefacts. Cells whose cytoplasm was contiguous (distance = 0) were then grouped together allowing for definition of individual cells and cell clusters.

#### Protein structural and functional analysis

The cDNAs of human EMB, BSG, and NPTN, as well as the W1 and W2 fragments of EMB (Figure S5) were cloned into GEX-2T vector and transformed to Escherichia coli strain BL21 Tuner (Novagen-Merck-EMD-Millipore). The GST-tagged proteins were produced and purified as described earlier (Sipilä et al., 2014; Tulla et al., 2001). Recombinant integrin α5β1 ectodomain was purchased from R&D Biosystems, and human plasma fibronectin, fibrinogen, laminin 1 and collagen I and collagen IV from Sigma Aldrich. Peptides P1 (N-terminal heparin binding fragment) and P2 (45kDa gelatin binding fragment) were purchased from Sigma-Aldrich (Catalog Number F9911 and F0162); P3 (Ser607-Pro1265), P4 (Glu1266-Pro1908), and P5 (Val1913-Glu2477) from R&D Systems (Catalog # 3225-FN, 3938-FN, and 3624-FN). Protein-protein interactions were studied with solid phase binding assays: a “ligand” coating (5 μg/cm^2^, overnight at +4°C) followed by blocking (Delfia Diluent, PerkinElmer, 1h at RT), adding a soluble “receptor” (Delfia Assay Buffer ± 2mM MgCl_2_ or MnCl_2_ or 5mM EDTA, 1h at RT), labeling (Europium-labeled anti-GST antibody, PerkinElmer, 1h at RT), and measuring (Delfia enhancement solution, Victor3 multilabel counter, PerkinElmer). The plates were washed 3 times with PBS between each step. In competition assays, a competitive component was added with a soluble “receptor”.

#### Structural modeling

Using the amino acid sequence of human embigin (UniProt KB code Q6PCB8) as bait with the Basic Local Alignment Search Tool (BLAST) at NCBI (http://blast.ncbi.nlm.nih.gov/), the non-redundant protein sequences database was searched for embigin sequences of varying identity and Protein Data Bank (PDB) for crystal structures to be used as templates for modeling. Two crystal structures of human basigin turned up as potential templates: PDB: 4U0Q (Wright et al., 2014) with E-value 4e-13 and 30.49 % percent identity and PDB: 3BH5 (Yu et al., 2008) with E-value 3e-04 and 26.32 % percent identity. The crystal structures of rat neuroplastin (PDB: 2WV3 (Owczarek et al., 2010)) and human neuroplastin (PDB: 6A69 (Gong et al., 2018)), which are close paralogs to basigin (Beesley et al., 2014), were superimposed on the structure of basigin using the program VERTAA (Johnson and Lehtonen, 2000) in the BODIL modeling environment (Lehtonen et al., 2004), generating a structure-based alignment. The embigin sequences from mouse (UniProt KB code P21995) and rat (UniProt KB code O88775) with 24.3% and 23.6% identity to basigin, respectively, were used to aid the alignment of embigin to the prealigned structure-based alignment of basigin and neuroplastin using the program MALIGN (Johnson et al., 1996) in BODIL to create the final alignment for modeling (Figure S6F). A set of ten models of embigin with basigin (PDB: 4U0Q) as template was created with MODELLER (Sali and Blundell, 1993), and the model with the lowest DOPE score was analyzed and compared to the crystal structures of basigin and neuroplastin by superimposition (Johnson and Lehtonen, 2000) in BODIL. The quality of the final model was assessed with ProSA web (Wiederstein and Sippl, 2007). PyMOL (version 1.6; Schrödinger, LLC) was used to prepare pictures of the 3D model and ESPript (Gouet et al., 1999) for the alignment picture.

### Quantification and statistical analysis

All the statistical analysis was performed with OriginLab or GraphPad Prism 6. Statistical significances were determined by unpaired two-tailed t-test. See figure legends for details of how n was determined. All the experiments were repeated ≥2. The length and width of SGs were quantified from tail whole mount images and nuclei density was determined from DAPI stained cross-sections using Fiji-ImageJ software. Sebocyte size was quantified from Herovici stained cross-sections with NDP.view2 software (Hamahatsu). Staining intensities were quantified by mean intensity or thresholding followed by a manual selection of the areas of interest using Fiji-Image: results were normalized to field, the size of area, or length of epithelial line as indicated in the Figure legends.

The average number of sebocytes per sebaceous gland (P and D, Figure S2) was approximated by assuming a SG to be an ellipsoid. The surface of the SG ellipsoid is covered by basal cells (P) and the volume filled by suprabasal cells. The area of a basal cell was calculated by using:

$$A=Nuclear\;density^{2}$$

and the volume of a suprabasal cell by using

$$V={\left(\sqrt{Average}\;cell\;size\right)}^{3}$$

The volume and area of SGs were approximated by using the volume and surface area formulas of an ellipsoid and average maximal length (a) and width (a = b) of SG:

$$V=\frac{4}{3}\pi abc\;A\approx 4\pi {\left(\frac{\left({\left(ab\right)}^{1.6}+{\left(ac\right)}^{1.6}+{\left(bc\right)}^{1.6}\right)}{3}\right)}^{1/1.6}$$

The length of hair follicles was quantified from tail whole mount images with Fiji-ImageJ. Tissue thickness was quantified from Herovici stained cross-sections with NDP.view2 software (Hamahatsu). The number of Immune cells was counted by Fiji-ImageJ from tail wholemount or cryosection images and normalized to epidermal area or field as indicated in the figure legend.

## Data Availability

• This paper analyzes existing, publicly available data. These accession numbers for the datasets are listed in the key resources table. • This paper does not report original code. • Any additional information required to reanalyze the data reported in this paper is available from the lead contact upon request.

## Supporting citations

The following reference appears in the supplemental information: Butler et al. (2018).

## Supplemental information

Document S1. Figures S1–S6 and Table S1

Document S2. Article plus supplemental information
