# Supplementary material for: Embigin is a fibronectin receptor that affects sebaceous gland differentiation and metabolism
Source: Dev Cell. 2022 Jun 20;57(12):1453–1465.e7. doi: 10.1016/j.devcel.2022.05.011 (PMC9616737; doi:10.1016/j.devcel.2022.05.011)
Supplement: Document S1. Figures S1–S6 and Table S1 [file mmc1.pdf]

Developmental Cell, Volume *57*

Supplemental information

Embigin is a fibronectin receptor that affects sebaceous gland differentiation and metabolism

Kalle Sipilä, Emanuel Rognoni, Johanna Jokinen, Mukul Tewary, Matteo Vietri Rudan, Salli Talvi, Ville Jokinen, Käthe M. Dahlström, Kif Liakath-Ali, Atefeh Mobasseri, Xinyi Du-Harpur, Jarmo Käpylä, Stephen L. Nutt, Tiina A. Salminen, Jyrki Heino, and Fiona M. Watt

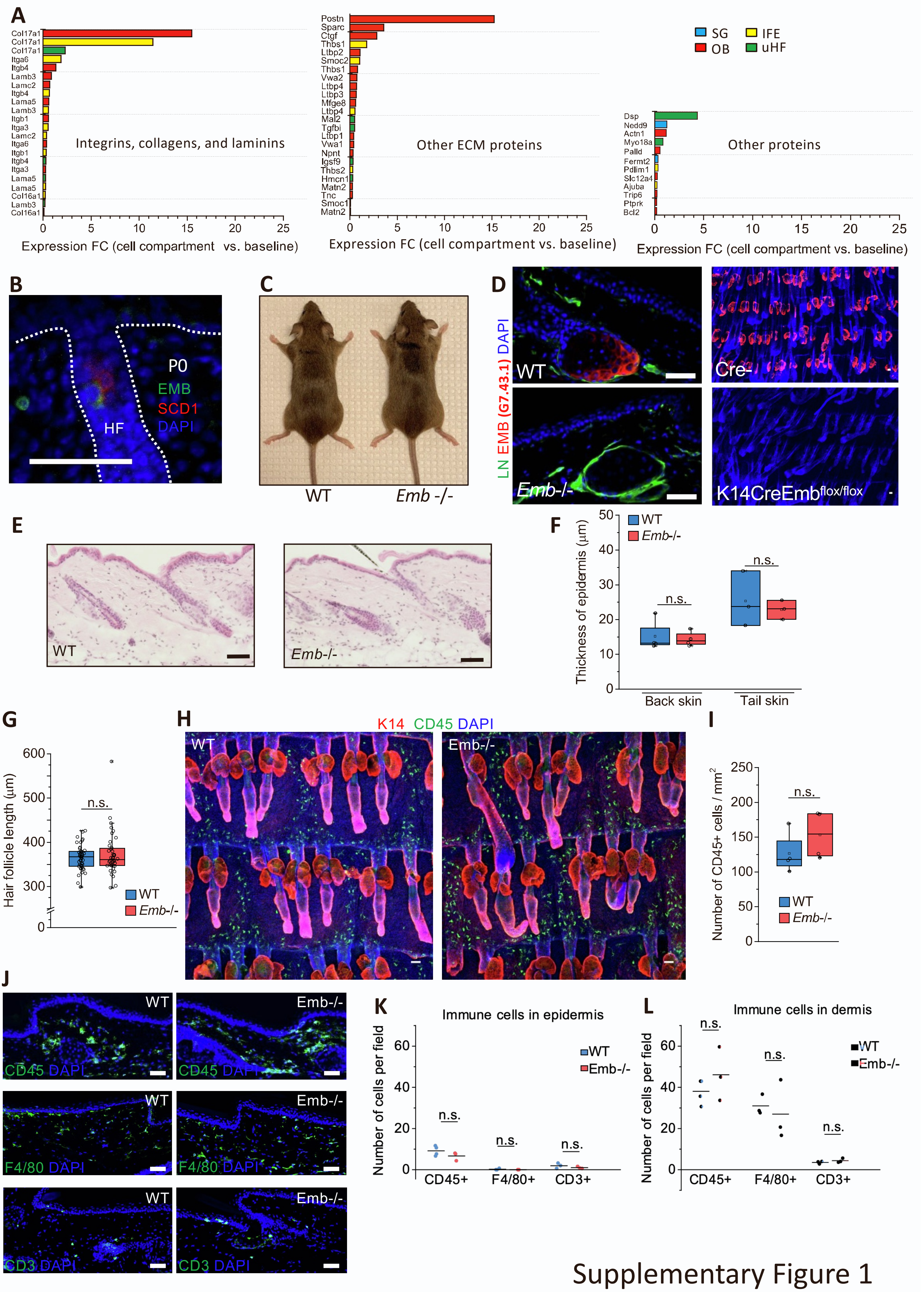

Supplementary Figure 1

Supplementary Figure 1. Characterization of Emb expression and knockout phenotype in skin, Related to Figure 1 and Figure 2.

(A): Epithelial marker genes, identified by Joost et al. (Joost et al., 2016), filtered by GO terms for cell-cell adhesion mediator (GO:0098632) and cell-matrix adhesion (GO:0007160), and divided to different graphs based on whether they are integrins, collagens, laminins, other ECM proteins or other that did not fall into these categories. SG: sebaceous gland; OB: outer bulge; IFE: interfollicular epidermis; uHF: upper hair follicle.

(B): Cryosection of back skin collected at P0 and labelled with antibodies against SCD1 (red) and EMB (green) with DAPI nuclear counterstain (blue).

(C): Images of 4-month-old female WT and *Emb-/-* mice.

(D): Cryosections of adult tail skin collected from WT and *Emb-/-* mice labelled with antibodies against pan-laminins (green) and EMB (red) with DAPI nuclear counterstain (blue) or tail wholemounts collected from Cre- or K14CreEmb$^{flox/flox}$ mice stained with EMB (red) and DAPI nuclear counterstain (blue).

(E): Hematoxylin & eosin staining of the back skin of adult WT and *Emb-/-* mice.

(F): Quantification of epidermal thickness in adult WT (n=4 back skin, n=3 tail skin) and *Emb*-/- (n=4 back skin, n=3 tail skin) mice.

(G): Quantification of telogen tail hair follicle length (WT, n=43; *Emb*-/-, n=40). The hair follicles were pooled from 4 adult WT and 4 adult *Emb*-/- mice.

(H): Telogen tail wholemounts from WT and *Emb*-/- mice labelled with antibodies against CD45 (green), K14 (red), with DAPI counterstain (blue).

(I): Quantification of CD45+ cells in tail wholemounts of adult WT (n=4) and *Emb*-/- (n=4) mice.

(J): Cryosection of tail skin stained with antibodies against immune cell markers CD45, F4/80, or CD3 (green) and DAPI counterstain (blue).

(K-L): Quantification of CD45+ (WT, n=4 mice; *Emb-/-,* n=3 mice), F4/80+ (WT, n=3 mice; *Emb-/-,* n=3 mice), or CD3+ (WT, n=3 mice; *Emb-/-,* n=3 mice) cells in epidermis (K) of dermis (L). 3-5 microscopy fields per mouse were quantified.

Two tailed t-test for independent means was used to determine the statistical significance in all cases. The length of scale bars is 50µm.

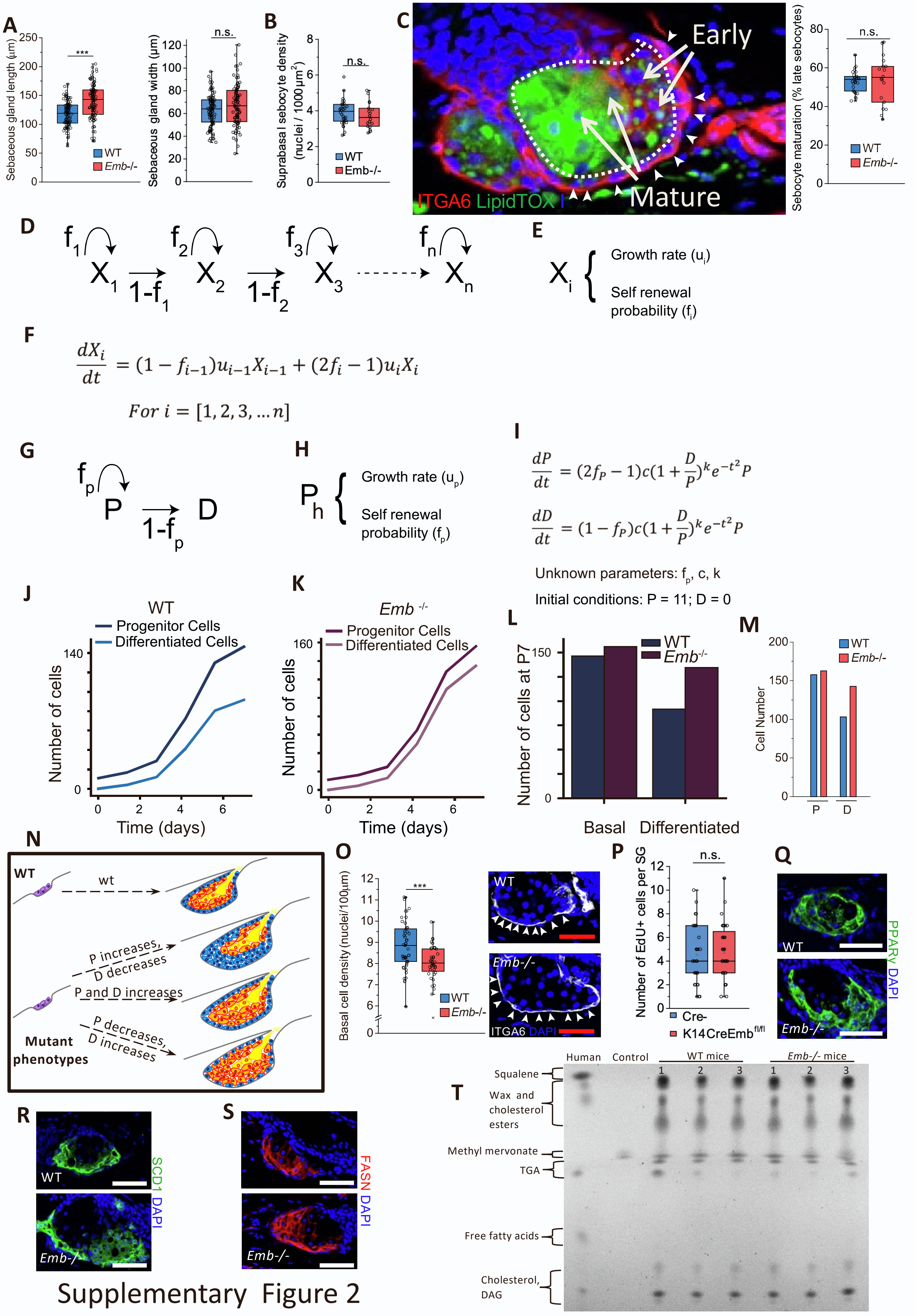

Supplementary Figure 2

Supplementary Figure 2. Characterization of Emb knockout sebaceous glands, Related to Figure 2.
(A): Quantification of SG length (WT, n=102; *Emb-/-,* n=111) and maximum width (WT, n=90; Emb-/-, n=86) in adult tail whole mounts. SGs were pooled from 4 WT and 4 *Emb-/-* mice.
(B): Quantification of suprabasal cell density in SGs (WT, n=30; *Emb-/-,* n=18). SGs were pooled from 6 WT and 4 *Emb-/-* mice.
(C): Adult WT SG labelled with LipidTOX (green), anti-ITGA6 (red) and DAPI (blue) and the quantification of ratio between early and late differentiated suprabasal cells in WT and *Emb*-/- SGs (WT, n=30; *Emb*-/-, n=18). SGs were pooled from 6 WT and 4 *Emb*-/- mice. Area surrounded by dotted line is suprabasal compartment. Arrowheads indicate basal cells. Arrows indicate suprabasal cells at early and late stages of differentiation.
(D-F): Theoretical description of stem cell compartments (given by Xi) giving rise to progressively specified cell types in a developing tissue. (D) Here, X1 represents the most primitive stem cell compartment, and Xn represents the final cell type that emerges in the developing tissue. (E) Each compartment (Xi) is associated with a specific growth/proliferation rate (given by ui) and the probability of these proliferating cells contributing to the self-renewal of the cell compartment (given by fi). Such a system may be described by a set of ordinary differential equations. If a cell compartment in such a system is terminally differentiated (i.e. not proliferating), it would have a growth/proliferation rate of u=0 and, as such, there would be no self-renewal or contribution to a subsequent cell compartment.
(G-I): Mathematical description of the developing SG as per the paradigm described in (D-F). (G) A simplified schematic representation of the development of the SG. The progenitor (basal) cells are the only proliferating cell compartment and they self-renew with a probability of fp, giving rise to the differentiated cells with a probability of (1-fp). The growth rate and self-renewal probability of the progenitor cells (H) would be the key regulators of the development of the SG. Here, we assumed that the growth rate would take the form of a Gaussian decay curve (u=αe-t2). Given the dramatic increase in size of differentiating cells, we reasoned that an increase in the differentiated cell compartment would require the proliferating basal cells to be able to rapidly respond and increase their proliferation/growth rate to accommodate for the increase in the volume of the sebaceous gland. Therefore, the proliferation rate of the progenitor (basal) cells is assumed to be a non-linear function of the ratio of the number of differentiated cells to the number of progenitor cells. This feedback is incorporated into the coefficient of the proliferation rate, where α=c(1+DP)k. Here, c, and k are constants, and along with fp they represent the unknowns of this system of ordinary differential equations. Taken together, the proliferation rate may be written as u=c(1+DP)ke-t2. A simplified mathematical representation of the developing sebaceous gland can therefore be represented as the ordinary differential equations shown in panel (I). The initial conditions for this system were based on Andersen et al. (Andersen et al., 2019) and the development of the sebaceous gland was assumed to start with 11 progenitor cells and no differentiated cells. Furthermore, the development time was chosen to be till P7 as per Andersen et al. (Andersen et al., 2019), after which the sebaceous gland is assumed to achieve homeostasis. The three unknown parameters of the derived set of ordinary differential equations were systematically trained to find the appropriate parameter set that would give output cell numbers for the progenitor and differentiated cell compartments of the developing wild type SG. The SG development for a 7-day period is shown in (J) for the parameter values fp = 0.7125, c = 1.27, and k = 6.
(J-M): Model predictions to account for the *Emb-/-* phenotype. We queried whether the model could predict the cell numbers in the progenitor and differentiated compartments of the *Emb*-/- mutant. Given that c and k are parameters that describe the inherent feedback system that regulate the proliferation of the progenitor (basal) cells, and we saw no changes in Ki67 expressing cells between the WT and *Emb-/-* SG (P, Figure 2F-G), we reasoned that these would remain unchanged in the absence of EMB. Since the only remaining parameter was the self-renewal probability (fp), we systematically tested different values and found that fp=0.675 closely recapitulated the cell numbers observed in the *Emb-/-* SG (L-M). The approximate number per average SG was calculated by assuming that each SG is an ellipsoid, the surface of which is covered by average size basal cells (O) and the volume formed by average volume sebocytes (B, Figure 2D).

(N): Schematic of how the number of progenitor cells (P) and differentiating cells (D) could account for the abnormal expansion of SG in different scenarios.
(O): Quantification of basal cell density in SGs (WT, n=49; *Emb-/-,* n=40). SGs were pooled from 4 WT and 4 *Emb-/-* mice. Representative images of WT and *Emb-/-* SGs with basement membrane (dotted line) and basal cell annotations (arrowheads) are shown.
(P): Quantification of EdU+ cell in SGs 4-6h after EdU injection (Day 0) (Cre-, n=30; K14CreEmb$^{flox/flox}$, n=32). SGs were pooled from 3 control and 3 *Emb-/-* mice.
(Q-S): Cryosections of wild type or Emb-/- tail skin stained with antibodies against proteins involved in lipid production: PPARγ (green) (Q), SCD1 (green) (R), or FASN (red) (S) and DAPI counterstain (blue).
(T): TLC showing lipid composition of sebum extractions from skin of 3 wild type, 3 *Emb-/-* mice and human skin.
Two tailed t-test for independent means was used to determine statistical significance. ***p<0.0005. Scale bars: 50µm.

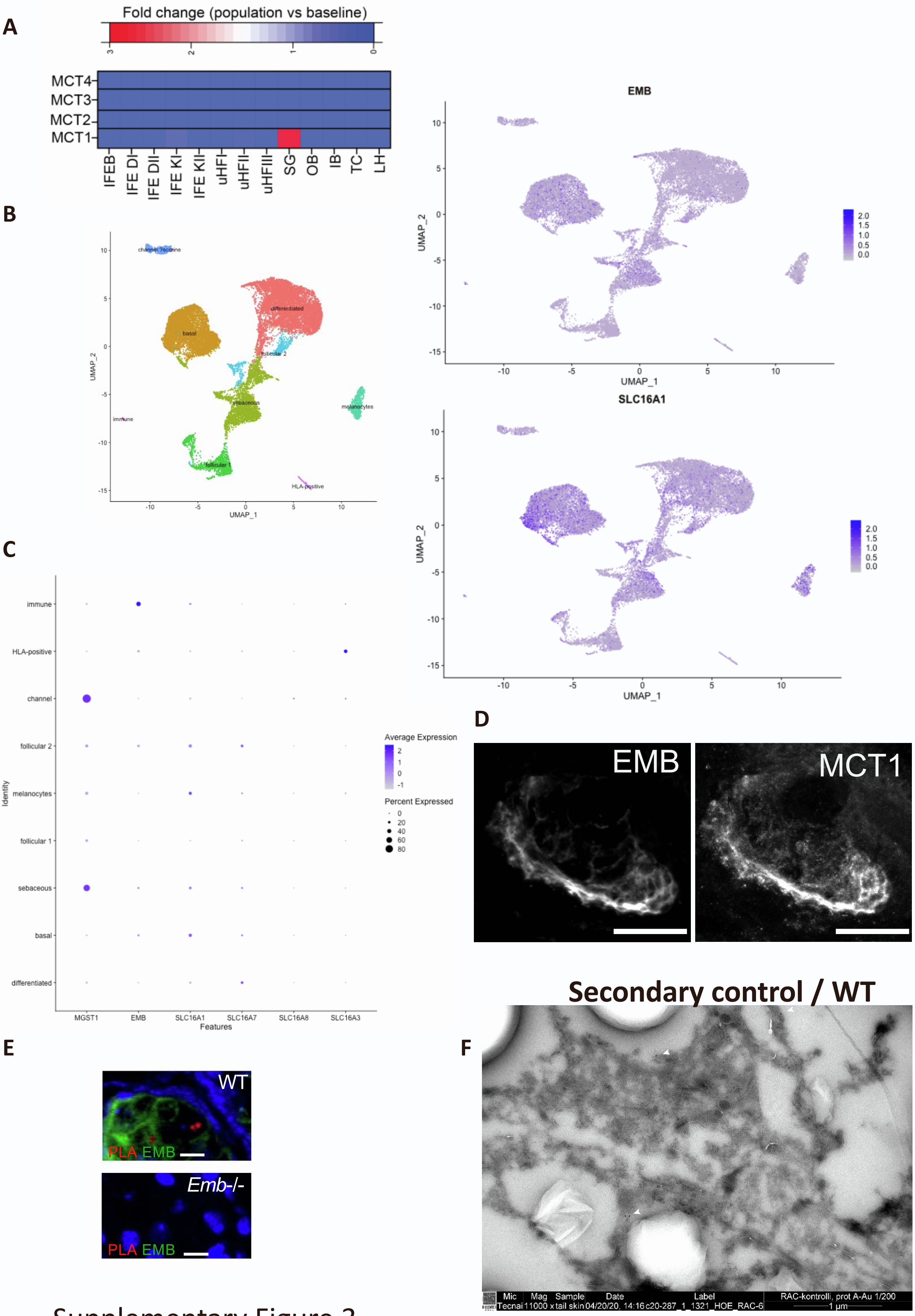

Supplementary Figure 3

Supplementary Figure 3. Emb interaction with monocarboxylate transporting in sebaceous glands, Related to Figure 3.

(A): Expression of different monocarboxylate transporters among the epithelial marker genes identified by Joost et al. (Joost et al., 2016); proteins/corresponding genes MCT1/*Slc16a1*, MCT2/*Slc16a7*, MCT3/*Slc16a8*, MCT4/*Slc16a3*.

(B-C): Expression of EMB and MCTs in human sebaceous glands. Publicly available single-cell RNA-seq data from human epidermis constituting over 92,000 cells was analyzed by using Seurat v3.0 (Butler et al., 2018; Cheng et al., 2018); proteins/corresponding genes MCT1/*SLC16A1*, MCT2/ *SLC16A7*, MCT3/ *SLC16A8*, MCT4/ *SLC16A3*.

(D): Cryosection of adult tail skin collected from WT and *Emb-/-* mice and labelled with antibodies against EMB and MCT1. Scale bars: 50µm.

(E): Proximity ligation assay (PLA, red) on wild type and *Emb-/-* tail skin cryosections stained with EMB (green) and DAPI (blue). The length of scale bars is 10µm.

(F): Transmission electron micrograph of adult SG labelled (secondary control) with Rabbit anti-Chicken (Jackson ImmunoResearch) and protein A-conjugated gold (10nm). Scale bars: 1µm. White arrowheads indicate gold particles.

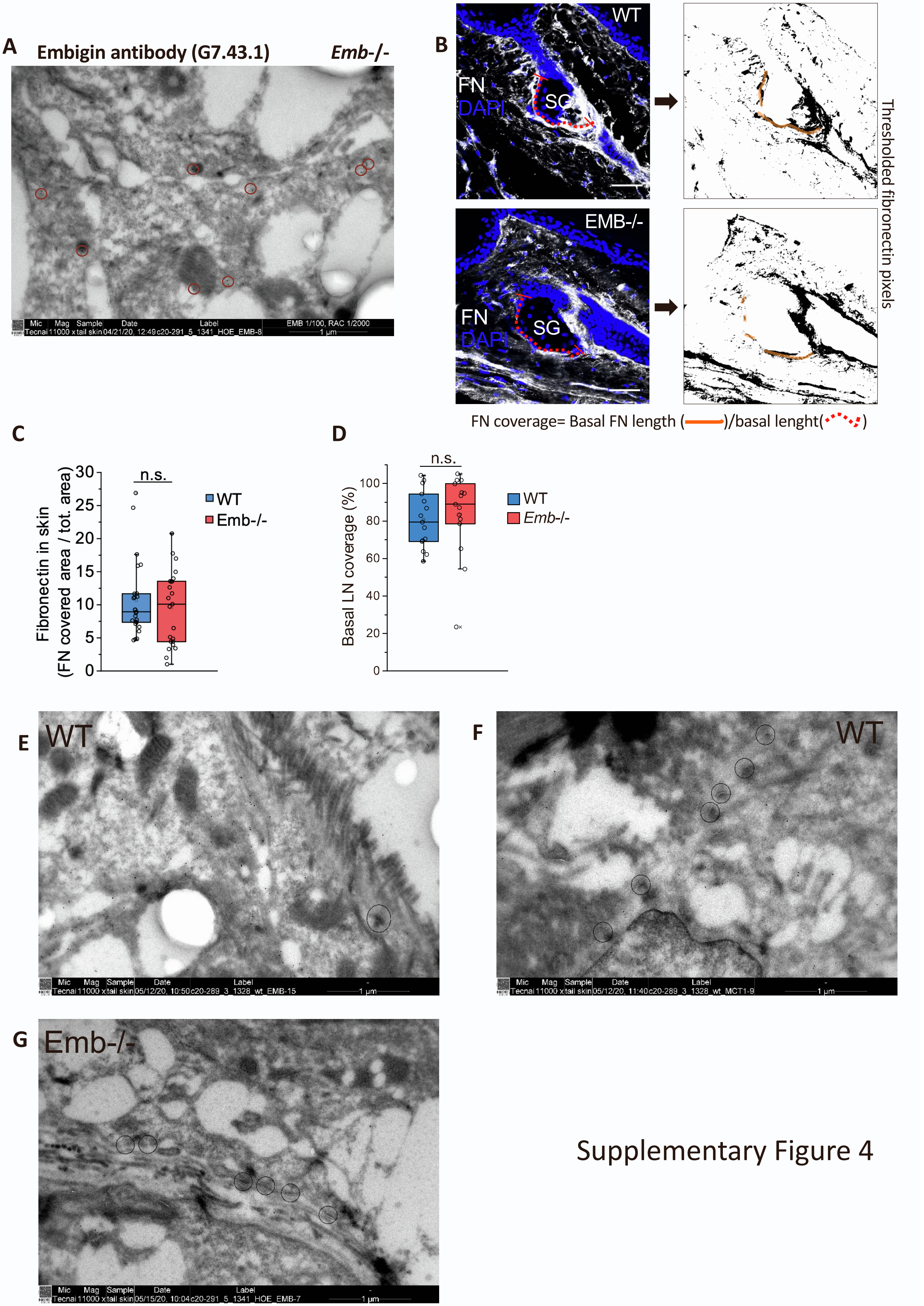

Supplementary Figure 4

Supplementary Figure 4. Association of Emb with ECM, Related to Figure 4.
(A): Transmission electron micrograph of *Emb-/-* mouse tail SG with EMB immunogold labelling. Scale bars: 1µm. Gold particles are denoted in the red circles.
(B): FN immunofluorescence labelling of SG (grey) with DAPI nuclear counterstain (blue) (left hand panel) showing the quantification pipeline of SG associated fibronectin. Scale bars: 50µm.
(C): Quantification of fibronectin in dermis (WT n=25 areas, *Emb-/-* n=23 areas). Dermal areas were pooled from 5 WT and 4 *Emb-/-* mice.
(D): Quantification of laminin in the basement membrane (WT, n=15; *Emb-/-*, n=15). SGs were pooled from 5 WT and 4 *Emb-/-* mice. The same pipeline was used for laminin and FN.
(E-F): Transmission electron micrograph of adult WT (E-F) or *Emb-/-* SG labelled with EMB (E, G) or MCT1 (F) antibodies and protein A -conjugated gold (10nm). Scale bars: 1µm. Hemidesmosomes are denoted in the black circles.
Two tailed t-test for independent means was used to determine statistical significance.

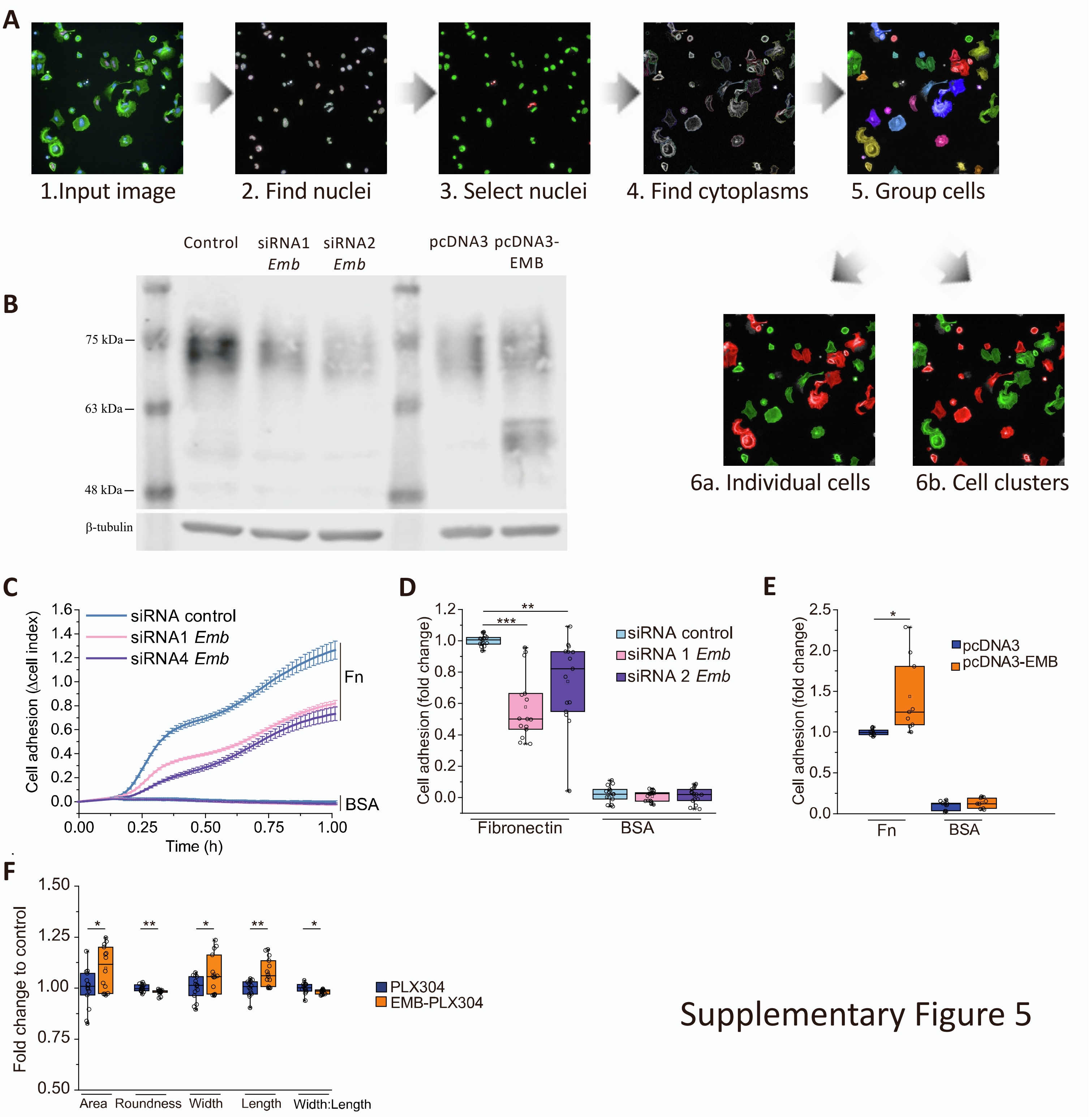

Supplementary Figure 5

Supplementary Figure 5. Emb increases cell adhesion to fibronectin, Related to Figure 5.
(A): Image analysis pipeline in Harmony software for identification of individual cells or cell clusters. Input images (1) were segmented to identify nuclei (2) that were subsequently filtered for size (> 85 μm^2 and < 1000 μm^2) and roundness (> 0.65) (3). The cell cytoplasm was then identified (4). Cells were grouped together if their cytoplasms were contiguous (distance = 0) (5) and classified as individual cells or cell clusters (6).
(B): Validation of overexpression and siRNA mediated silencing of EMB. Western blot was stained with G7.43.1 antibody against EMB and β-tubulin as a loading control. Molecular mass markers are shown.
(C-D): siRNA silencing of Emb in mouse keratinocytes followed by a real time adhesion measurement (xCELLigence, Roche). The average ± SD of 4 independent measurements is shown in (C) and the 1h time point in (D). n=12 replicates of each condition from 3 independent experiments normalized to the average of controls in each experiment.
(E): Effect of overexpression of EMB in mouse keratinocytes measured by a real time adhesion measurement (xCELLIgence) 1h after plating on FN or BSA. n=12 replicates of each condition from 3 independent experiments normalized to the average of controls in each experiment.
(F): Operetta (PerkinElmer) measurement of cell morphological parameters in mouse keratinocyte overexpressing EMB (EMB-PLX304) or the plasmid control (PLX304) 2h after plating on FN without the presence of serum. 14 replicates of each condition in 3 independent experiments normalized to the average of controls in each experiment are shown.
Two tailed t-test for independent means was used to determine statistical significance. ***$p<0.0005$, ** $p<0.005$, * $p<0.05$.

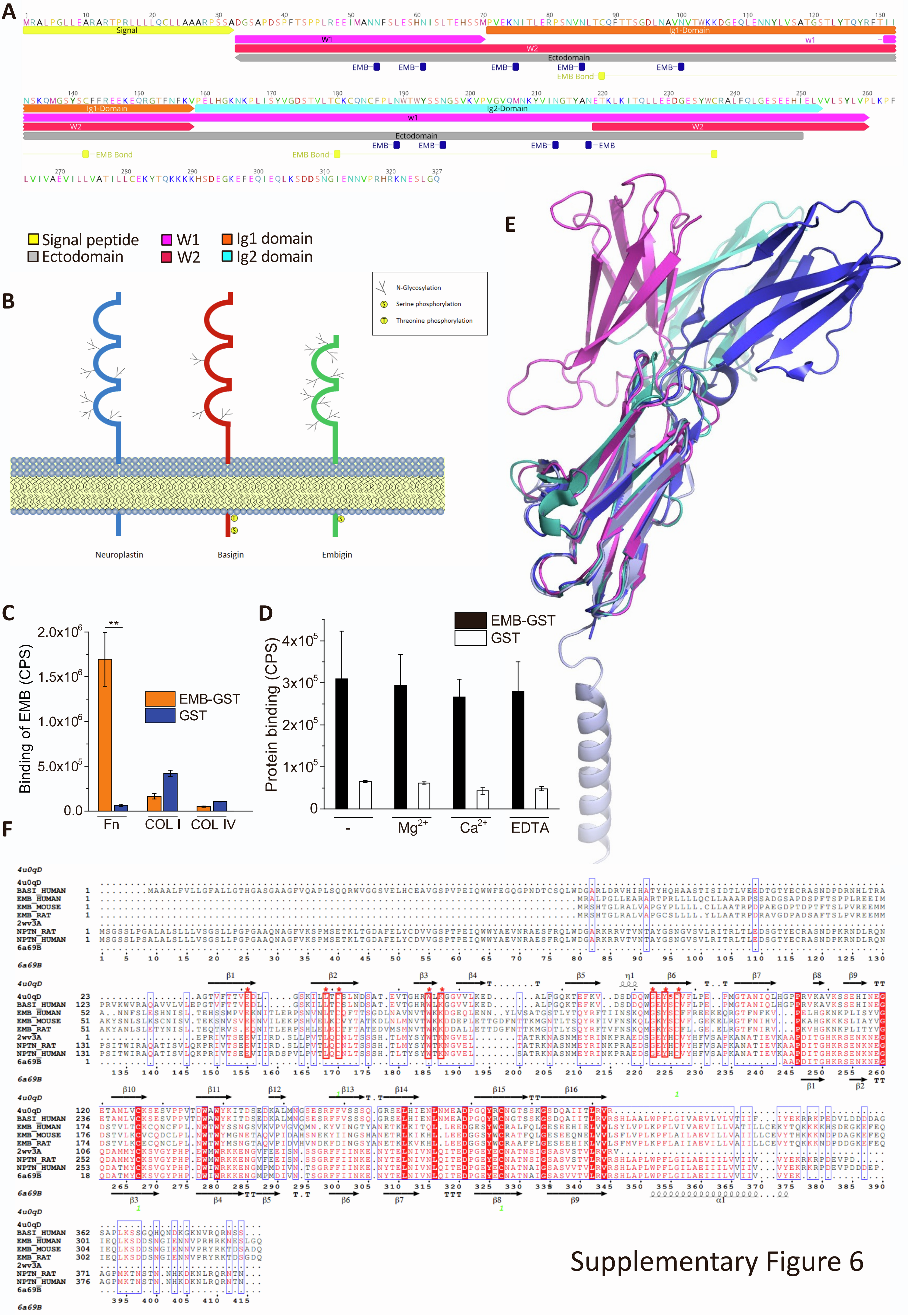

Supplementary Figure 6

Supplementary Figure 6. Characterization of Emb binding to fibronectin, Related to Figure 6.
(A): The amino acid sequences of the recombinant proteins aligned with full lenght EMB (UniProtKB: Q6PCB8)
(B): immunoglobulin subfamily of basigin (UniProtKB: - P35613), neuroplastin (UniProtKB: Q9Y639), and embigin.
(C): Binding of recombinant EMB-GST or GST (200nM) to fibrillar collagen (COL I) and basement membrane collagen in the native conformation (COL IV). Averages of 3 replicates ±S D are shown.
(D): Binding of recombinant EMB-GST or GST (200nM) to fibronectin in the presence of 2mM metal ions or 15mM EDTA. Averages of 3 replicates ± SD are shown.
(E): Comparison of human basigin (deep teal), rat neuroplastin (blue, PDB: 2wv3B) and domain 2 and the transmenbrane helix of human neuroplastin (light blue, PDB: 6a69) crystal structures with the embigin model (magenta). The more conserved domain 2 of each structure is superimposed to show the different angles between the two domains in each structure.
(F): The sequence alignment of embigin, basigin, and neuroplastin (see "Star Methods").
Two tailed t-test for independent means was used to determine statistical significance. ** $p<0.005$.

Parameter	Value
f_p	0.7125
c	1.27
k	6
Initial Conditions	P = 11; D = 0
Development start	P2
Development end (start of homeostasis)	P9

Supplementary table 1. Final parameter values for mathematical modeling, related to STAR methods.
